# Structural basis for the endoribonuclease activity of the type III-A CRISPR-associated protein Csm6

**DOI:** 10.1261/rna.054098.115

**Published:** 2016-03

**Authors:** Ole Niewoehner, Martin Jinek

**Affiliations:** Department of Biochemistry, University of Zurich, Zurich CH-8057, Switzerland

**Keywords:** CRISPR, Cas protein, ribonuclease, Csm6, crRNA, HEPN domain

## Abstract

Prokaryotic CRISPR–Cas systems provide an RNA-guided mechanism for genome defense against mobile genetic elements such as viruses and plasmids. In type III-A CRISPR–Cas systems, the RNA-guided multisubunit Csm effector complex targets both single-stranded RNAs and double-stranded DNAs. In addition to the Csm complex, efficient anti-plasmid immunity mediated by type III-A systems also requires the CRISPR-associated protein Csm6. Here we report the crystal structure of Csm6 from *Thermus thermophilus* and show that the protein is a ssRNA-specific endoribonuclease. The structure reveals a dimeric architecture generated by interactions involving the N-terminal CARF and C-terminal HEPN domains. HEPN domain dimerization leads to the formation of a composite ribonuclease active site. Consistently, mutations of invariant active site residues impair catalytic activity in vitro. We further show that the ribonuclease activity of Csm6 is conserved across orthologs, suggesting that it plays an important functional role in CRISPR–Cas systems. The dimer interface of the CARF domains features a conserved electropositive pocket that may function as a ligand-binding site for allosteric control of ribonuclease activity. Altogether, our work suggests that Csm6 proteins provide an auxiliary RNA-targeting interference mechanism in type III-A CRISPR–Cas systems that operates in conjunction with the RNA- and DNA-targeting endonuclease activities of the Csm effector complex.

## INTRODUCTION

In many bacteria and most archaea, CRISPR–Cas systems (for clustered regularly interspaced short palindromic repeats—CRISPR-associated) provide adaptive and heritable immunity against mobile genetic elements such as viruses and plasmids (Barrangou et al. 2007; Marraffini and Sontheimer 2008, 2010; Wiedenheft et al. 2012; Sorek et al. 2013; van der Oost et al. 2014). These RNA-guided genome defense systems typically consist of an array of short repeats intercalated with invader-derived spacer sequences, and an operon containing several CRISPR-associated (*cas*) genes encoding the molecular machinery involved in spacer acquisition, guide RNA processing, and target interference. Transcription of CRISPR spacer-repeat arrays and subsequent processing of the precursor transcripts yields individual CRISPR RNAs (crRNAs) (Brouns et al. 2008; Carte et al. 2008; Hale et al. 2008; Haurwitz et al. 2010). These crRNA guides in turn associate with Cas proteins in effector complexes in which they mediate target detection by Watson–Crick base-pairing interactions (Brouns et al. 2008; Hale et al. 2009; Jore et al. 2011). Five types of CRISPR–Cas systems (types I–V) have been identified, each having distinct composition of the *cas* gene operon and distinct mechanisms of crRNA biogenesis and crRNA-guided interference (Makarova et al. 2011a, 2015).

In type I CRISPR–Cas systems, multiple Cas proteins assemble with a mature crRNA in a large multisubunit complex, termed Cascade, that facilitates recognition of double-stranded DNA (dsDNA) targets (Brouns et al. 2008). Upon target binding, the Cascade complex recruits the type I-specific helicase/exonuclease Cas3 that degrades the target DNA in a processive manner (Brouns et al. 2008; Beloglazova et al. 2011; Sinkunas et al. 2011; Westra et al. 2012). In contrast, type II and type V systems target dsDNA by means of single effector proteins Cas9 and Cpf1, respectively, that function as RNA-guided DNA endonucleases (Deltcheva et al. 2011; Zetsche et al. 2015). Cas9 associates with a dual-RNA guide structure consisting of a crRNA and a trans-activating CRISPR RNA (tracrRNA) and cleaves dsDNA within a target sequence complementary to a 20-nucleotide (nt) guide segment in the crRNA (Deltcheva et al. 2011; Gasiunas et al. 2012; Jinek et al. 2012). Type III CRISPR–Cas systems are defined by the signature protein Cas10 and, in analogy with type I systems, rely on multisubunit crRNA–Cas protein complexes for target recognition (Marraffini and Sontheimer 2008; Hale et al. 2009; Makarova et al. 2011a). The two type III CRISPR–Cas system subtypes, type III-A and III-B, have different subunit compositions and are thought to have different target specificities. The type III-B effector complex (Cmr complex), containing proteins Cmr1, Cmr2/Cas10, and Cmr3–6, has been shown to bind and cleave single-stranded RNA (ssRNA) targets in vitro and in vivo (Hale et al. 2009, 2012; Zhang et al. 2012; Staals et al. 2013; Zebec et al. 2014). Within the Cmr complex, the Cmr4 subunits mediate cleavage of ssRNAs complementary to the spacer-derived part of the crRNA at discrete 6-nt intervals in a 5′ ruler-dependent manner (Staals et al. 2013; Benda et al. 2014; Hale et al. 2014; Ramia et al. 2014; Zhu and Ye 2015). Type III-A effector complexes (Csm complexes) are composed of a crRNA and Cas proteins Csm1/Cas10 and Csm2–5 (Marraffini and Sontheimer 2008). The type III-A CRISPR–Cas system is believed to target DNA based on its ability to interfere with plasmid transformation and conjugation in *Staphylococcus epidermidis* (Marraffini and Sontheimer 2008; Hatoum-Aslan et al. 2013, 2014). DNA targeting by the *S. epidermidis* CRISPR–Cas system requires active transcription of the target DNA, thereby enabling conditional tolerance of temperate phages (Goldberg et al. 2014; Peng et al. 2015). Purified Csm complexes from *Streptococcus thermophilus* and *Thermus thermophilus* cleave ssRNA in vitro by a mechanism similar to that of the Cmr complex (Staals et al. 2014; Tamulaitis et al. 2014). The *S. epidermidis* Csm complex has recently been shown to harbor two independent endonuclease activities (Samai et al. 2015). The complex is capable of cleaving ssRNA targets complementary to the crRNA; additionally, the complex cleaves double-stranded target DNAs within the nontemplate strand in a transcription-dependent manner. Whereas the RNA cleavage activity of the Csm complex is mediated by the Csm3 subunits (homologous to Cmr4 subunits of the Cmr complex), DNA cleavage requires an intact palm polymerase domain in the Csm1/Cas10 subunit (Samai et al. 2015). These results collectively suggest that type III-A systems target both RNA and DNA, which is further underscored by experiments showing that type III-A systems are capable of restricting both DNA and RNA bacteriophages in vivo (Goldberg et al. 2014; Tamulaitis et al. 2014; Samai et al. 2015).

*csm*6 genes are frequently associated with type III CRISPR–Cas systems (Makarova et al. 2011b). The Csm6 protein is distantly related to the type I-A associated protein Csa3, which has been suggested to function as a transcription factor that controls Cas protein expression (Lintner et al. 2011). In contrast to Csa3, in vivo experiments performed in *S. epidermidis* have shown that Csm6 is not required for Csm complex expression or assembly, suggesting that Csm6 does not function as a transcriptional regulator (Hatoum-Aslan et al. 2014). Instead, Csm6 is essential for crRNA-guided anti-plasmid interference, although it is not an integral component of the Csm effector complex (Hatoum-Aslan et al. 2013, 2014). Both Csm6 and its homologous protein Csx1 are members of the COG1517 superfamily and are found in four distinct Cas protein families (Anantharaman et al. 2013). Csx1 has been shown to be required for the interference activity of a type III-B CRISPR–Cas system in *Sulfolobus islandicus* (Deng et al. 2013). Both Csm6 and Csx1 proteins share an overall architecture defined by an N-terminal CARF domain (CRISPR-associated Rossman fold) and a C-terminal HEPN domain (higher eukaryotes and prokaryotes nucleotide-binding domain) that contains a conserved R-X_4-6_-H motif (Anantharaman et al. 2013; Kim et al. 2013). Domains belonging to the HEPN superfamily often exhibit ribonuclease activity and are commonly found in prokaryotic toxin–antitoxin (T–A) and abortive infection (Abi) defense systems, as well as in KEN (kinase-extension nuclease) domain-containing eukaryotic ribonucleases such as RNase L and Ire1 (Anantharaman et al. 2013). Due to the high conservation of the putative HEPN domain active site in Csm6 proteins, it is conceivable that these proteins function as ribonucleases.

In this study, we sought to shed light on the biochemical function of Csm6 proteins in type III-A CRISPR–Cas systems. We report the crystal structure of *T. thermophilus* Csm6 (TtCsm6) at 2.3 Å resolution and show that the protein is a ssRNA-specific endoribonuclease. The HEPN domains in the TtCsm6 dimer form a composite ribonuclease active site whose architecture and function is likely conserved in other Csm6 proteins. These results suggest that besides the intrinsic RNA cleavage activity of the Csm effector complex mediated by its Csm3 subunits, type III-A CRISPR–Cas systems harbor an additional ribonuclease module—Csm6—whose activity may play an important role in the interference mechanism.

## RESULTS AND DISCUSSION

### TtCsm is an ssRNA-specific endoribonuclease

**FIGURE 1.**
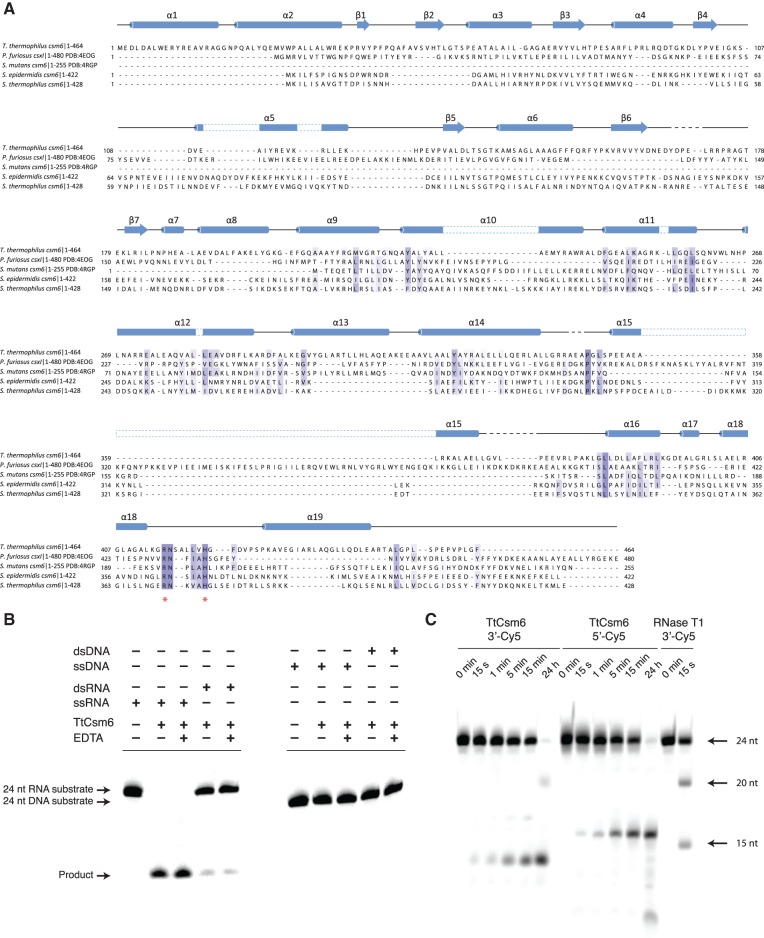
*Thermus thermophilus* Csm6 (TtCsm6) is a single-strand-specific endoribonuclease. (*A*) Multiple sequence alignment of Csm6 and Csx1 proteins from *T. thermophilus* (TtCsm6, GI:55978335), *S. epidermidis* (SeCsm6, GI:488416649), *S. mutans* (SmCsm6, GI:24379650), *S. thermophilus* (StCsm6, GI:585230687), and *P. furiosus* Csx1 (PfCsx1, GI:33359545), performed using ClustalOmega (McWilliam et al. 2013). HEPN domain active site residues are marked with asterisks. Secondary structure elements of TtCsm6 are indicated *above* the sequences. Disordered amino acid residues are indicated with dashed lines. (*B*) Nuclease activity assays performed with single- (ss) and double-stranded (ds) RNA and DNA oligonucleotide substrates. The 24-nt substrates have identical sequences and carry a Cy5 fluorophore group covalently attached to the 3′-terminus. Double-stranded substrates were prepared by annealing an unlabeled complementary strand to the respective single-stranded substrate. TtCsm6 (400 nM final concentration) was incubated with substrates (200 nM) at 37°C for 1 h. Cleavage products were analyzed by electrophoresis on a 15% denaturing polyacrylamide gel and detected using a fluorescence gel scanner. (*C*) Nuclease activity assays performed using TtCsm6 and identical ssRNA oligonucleotide substrates (24 nt) labeled with Cy5 at the 5′ or 3′ ends. The assay was performed as in *B*. A control digest with RNase T1 was used to generate RNA fragments of defined size, as indicated.

The HEPN protein superfamily has recently been expanded by the inclusion of four distinct Cas protein families, two of which are classified as Csm6 proteins and two as Csx1 proteins (Anantharaman et al. 2013). HEPN domains occur across all domains of life and are characterized by the presence of a conserved motif conforming to the consensus sequence R-X_4-6_-H (Anantharaman et al. 2013). The domains are frequently found in ribonucleases and in several of these the R-X_4-6_-H motif has been shown to be required for catalytic activity (Davidov and Kaufmann 2008; Lee et al. 2008; Han et al. 2014; Huang et al. 2014). The nuclease activity of Csm6 proteins has not been tested to date, although *Pyrococcus furiosus* Csx1 was previously shown to be a nucleic acid-binding protein (Kim et al. 2013). We examined the HEPN domains in Csm6 and Csx1 proteins by performing a multiple sequence alignment of representative orthologs from organisms that have been investigated extensively in the CRISPR–Cas field (Fig. 1A). The overall sequence identity in pairwise alignments was rather low, ranging from ∼9% to ∼28%. However, the HEPN domain R-X_4-6_-H motif was strictly conserved in all examined sequences, pointing to its conserved functional role in Csm6 and Csx1 proteins, and therefore hinting that these proteins function as ribonucleases. To test this hypothesis, we expressed and purified recombinant *T. thermophilus* Csm6 protein (TtCsm6) and probed its nuclease activity using fluorescently labeled RNA and DNA oligonucleotides. A randomly selected RNA oligonucleotide sequence was efficiently cleaved by TtCsm6 at 37°C to yield discrete shorter oligonucleotide products (Fig. 1B). In contrast, a dsRNA duplex containing the same oligonucleotide sequence was largely refractory to cleavage; the trace amount of product generated by TtCsm6 was possibly due to duplex strand dissociation during the cleavage reaction. Neither ssDNA nor dsDNA oligonucleotides of the same nucleotide sequence were cleaved by TtCsm6, indicating that the protein is a ribonuclease. Furthermore, ssRNA cleavage by TtCsm6 was not perturbed by the addition of EDTA, indicating that the activity of TtCsm6 does not require divalent metals. To characterize the cleavage activity of TtCsm6 further, we performed a nuclease activity assay using ssRNA oligonucleotide substrates that were fluorescently labeled at either the 3′- or 5′-end. In both cases, incubation of the substrate with TtCsm6 resulted in the formation of shorter oligonucleotide products (Fig. 1C). This suggests that TtCsm6 harbors an endoribonuclease, rather than exonuclease activity because covalent modification of neither the 3′ nor the 5′ terminus with the Cy5 fluorophore inhibited RNA cleavage. To examine the sequence/base specificity of TtCsm6, we analyzed its activity against synthetic 12-mer homo-oligonucleotides (U_12_, A_12_, and C_12_). Incubation of all three substrates with TtCsm6 resulted in the formation of anomalously migrating short oligonucleotide products, including the terminal dinucleotides (Supplemental Fig. 1; Killelea et al. 2014). This suggests that the enzymatic activity of TtCsm6 is largely sequence-unspecific and does not appear to be selective for a specific substrate or product length. Taken together, these experiments show that TtCsm6 is a divalent metal-independent, ssRNA-specific, endoribonuclease. This is consistent with the catalytic activities of other HEPN-domain ribonucleases such as Ire1, RNase L, and the tRNA anticodon RNases PrrC and RloC, which are all metal-independent enzymes (Davidov and Kaufmann 2008; Lee et al. 2008; Meineke and Shuman 2012; Han et al. 2014; Huang et al. 2014).

To probe the chemistry of Csm6-catalyzed RNA hydrolysis, we digested the 3′-end labeled oligonucleotide with TtCsm6 and subsequently incubated the cleavage products with T4 polynucleotide kinase (PNK) in the presence of ATP. Treatment with T4 PNK resulted in a marked shift in electrophoretic mobility, indicating that the 3′ products of Csm6-catalyzed phosphodiester bond hydrolysis carry a free 5′-hydroxyl group (Supplemental Fig. 2). This suggests that RNA substrate cleavage by Csm6 yields products containing a 5′-hydroxyl and a 2′–3′ cyclic phosphate groups, as shown for other HEPN domain RNases (Amitsur et al. 1987; Gonzalez et al. 1999; Davidov and Kaufmann 2008).

### The crystal structure of TtCsm6 reveals a dimeric architecture

To shed light on the molecular architecture of Csm6 and the functional organization of its constituent domains, we determined the three-dimensional structure of TtCsm6 by X-ray crystallography. TtCsm6 crystallization was dependent on the presence of Ni^2+^ ions, which allowed us to solve the structure using single-wavelength anomalous diffraction (SAD) phasing. The atomic model was refined at a resolution of 2.30 Å with *R*_work_ of 21.9% and *R*_free_ of 24.9% (Table 1). The protein crystallized as a homodimer, consistent with size-exclusion chromatography data indicating that the protein is dimeric in solution (Supplemental Fig. 3).

**Table 1.**
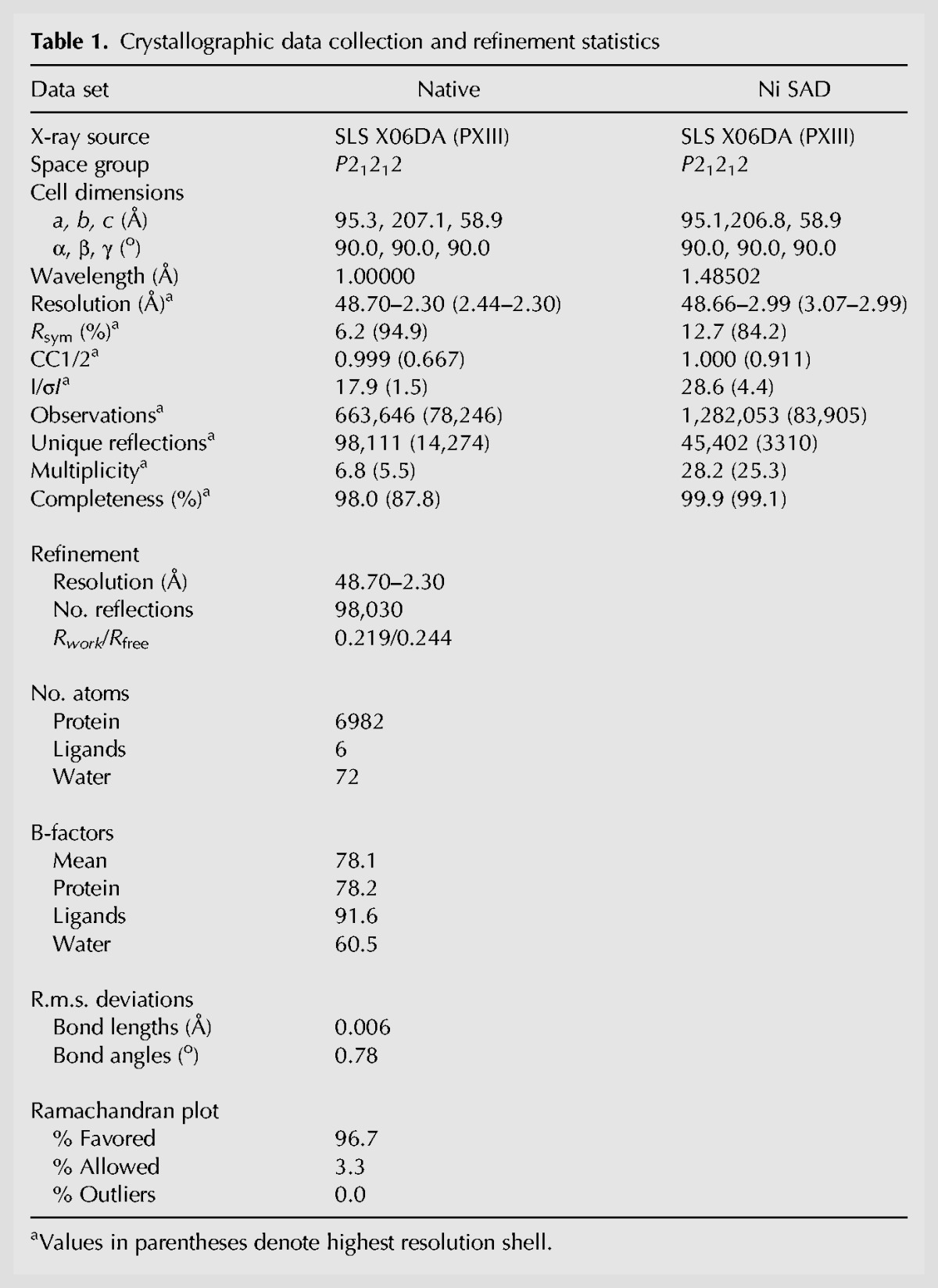
Crystallographic data collection and refinement statistics

**FIGURE 2.**
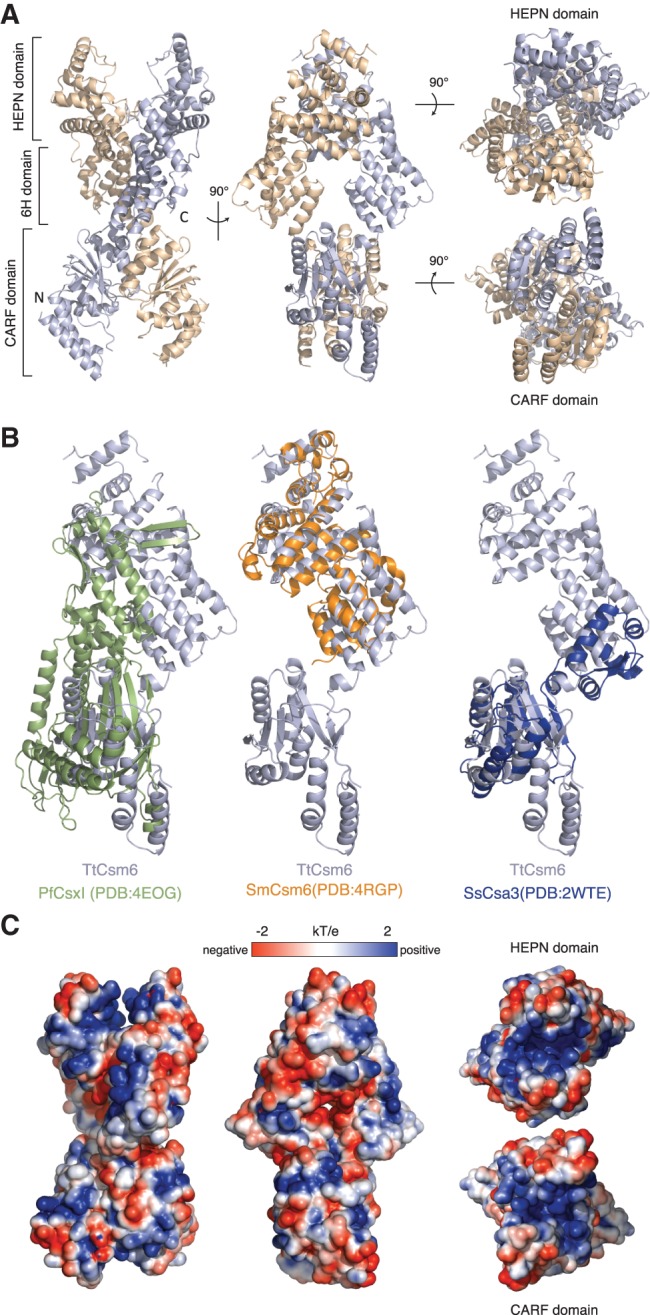
TtCsm6 is a helical homodimer containing N-terminal CARF and C-terminal HEPN domains. (*A*) Overall architecture of the TtCsm6 dimer shown in four orientations. (*B*) Comparison of the three-dimensional structures of COG1517 family proteins: *P. furiosus* Csx1 (PfCsx1, PDB ID 4EOG), *S. mutans* Csm6 (SmCsm6, PDB ID 4RGP), and *S. solfataricus* Csa3 (SsCsa3, PDB ID 2WTE). Structural superpositions were performed using DALI (Hasegawa and Holm 2009). (*C*) Surface representation of the TtCsm6 dimer colored according to electrostatic surface potential. The molecule is displayed in the same orientations as in *A*.

The TtCsm6 homodimer resembles an elongated X-shape with overall dimensions of ∼120 Å × 70 Å × 60 Å. The polypeptide chains of the two protomers are arranged in a parallel, head-to-head fashion, twisting around each other in a right-handed double helix (Fig. 2A). The N-terminal region of TtCsm6 (residues 1–184) comprises a CRISPR-associated Rossman fold (CARF) domain and consists of five parallel and two anti-parallel β-strands flanked by pairs of α-helices on either side of the central β-sheet. The remainder of the polypeptide, connected to the CARF domain via a short linker, is almost completely α-helical and is composed of two domains. The middle region of the TtCsm6 polypeptide chain (residues 191–292) consists of five α-helices and forms a right-handed solenoid domain previously denoted as the 6H domain (Makarova et al. 2014). The C-terminal region of TtCsm6 (residues 293–450) comprises eight α-helices that form the HEPN domain. The C-terminus of the Csm6 polypeptide chain (residues 451–464) folds back onto the 6H domain of the same protomer.

The overall domain architecture of TtCsm6 is similar to other members of the COG1517 superfamily whose structures have been determined. Csx1 proteins from *P. furiosus* (PfCsx1, PDB ID: 4EOG) and *Sulfolobus solfataricus* (SsCsx1, PDF ID: 2I71) contain both the N-terminal CARF and the C-terminal HEPN domains but lack the middle 6H region. Structural alignment performed using the DALI server (Holm and Rosenström 2010) revealed that the N-terminal CARF domain of TtCsm6 superimposes with the CARF domains of PfCsx1 and SsCsx1 with a root mean square deviation (rmsd) of 2.7 Å (over 124 Cα atoms) and 3.1 Å (over 127 Cα atoms), respectively (Fig. 2B). In contrast, the C-terminal HEPN domains of TtCsm6, PfCsx1, and SsCsx1 could not be superimposed. This is largely due to a structural rearrangement of the HEPN domain in Csx1 proteins due to the insertion of a β-hairpin, as noted previously (Anantharaman et al. 2013). *Streptococcus mutans* Csm6 (SmCsm6, PDB ID: 4RGP) superimposes with an rmsd of 4.0 Å over 198 Cα atoms. In this case, the structural homology is restricted to the C-terminal HEPN domain because SmCsm6 lacks the N-terminal CARF domain (Figs. 1A, 2B). The CARF domain of TtCsm6 also displays strong structural homology with the putative transcriptional regulator Csa3 from *S. solfataricus*, superimposing with an rmsd of 2.8 Å over 120 Cα atoms (Fig. 2B).

Dimerization of TtCsm6 buries ∼2800 Å^2^ of solvent-accessible surface area per chain and is mediated by two dimer interfaces, one involving the N-terminal CARF domains and the other comprising both the 6H and HEPN domains. The CARF domain dimerization interface is centered on helix α7 and involves largely hydrophobic contacts (Supplemental Fig. 4). The 6H and HEPN domains dimerize via hydrophobic interactions involving amino acid residues provided by helices α12 and α14 and by the loop connecting helices α18 and α19, which contains the R-X_4-6_-H motif (Supplemental Fig. 4). Overall, the architecture of the TtCsm6 dimer is similar to the crystallographic dimers of PfCsx1 and SsCsx1 (Kim et al. 2013). However, the hydrophobic nature of the dimerization interface is the likely reason for TtCsm6 forming a stable homodimer in solution, in contrast to PfCsx1 and SsCsx1, which are both monomeric in solution. In this respect, TtCsm6 resembles Csa3, which also forms a stable homodimer (Lintner et al. 2011).

### The TtCsm6 dimer features two putative nucleotide-binding sites

Examination of the electrostatic surface potential of TtCsm6 revealed the presence of two large positively charged patches located at opposite ends of the dimer, both centred on the dyad axis of the dimer (Fig. 2C). The patches are found in clefts formed by the respective dimerization of the CARF and HEPN domains. To analyze the phylogenetic conservation of these clefts, we identified the 50 closest nonredundant orthologs of TtCsm6 using PSI-BLAST (Altschul et al. 1997) and aligned their sequences using COBALT (Supplemental Data File 1; Papadopoulos and Agarwala 2007). Mapping amino acid sequence conservation onto the molecular surface of TtCsm6 using the Consurf server (Landau et al. 2005) shows that in contrast to other regions of the Csm6 dimer, both clefts are highly conserved, hinting at their functional importance (Supplemental Fig. 5). The CARF domain is a variant of the Rossman fold, which is often found in nucleotide-binding enzymes (Hanukoglu 2015). CARF domains lack the canonical G-X-G-X-(G/A) motif, suggesting that they do not bind NAD(P)H or FADH_2_. Instead, they harbor a conserved (D/N)-X-(S/T)-X_3_-(R/K) motif that maps to the canonical ligand-binding face of the Rossmann fold (Makarova et al. 2014). In TtCsm6, this motif (Asp131–Lys137) is partially exposed and forms the base of the deep cavity spanning the CARF dimer interface (Supplemental Fig. 5). Although this motif contributes to dimer formation, its surface accessibility points to an additional function in ligand binding. It has previously been proposed that CARF domain proteins such as Csa3 might recognize and be allosterically regulated by nucleotides or nucleotide-derived metabolites (Lintner et al. 2011). The evolutionary conservation of the CARF domain cleft in TtCsm6 and its strong electropositive surface potential collectively suggest that this might also be the case for Csm6 and Csx1 proteins. The concave surface of the HEPN domain dimer has previously been implicated in nucleic acid binding in other Csm6/Csx1 proteins. PfCsx1 binds both dsDNA and dsRNA using arginine side chains projecting into the HEPN domain cleft (Kim et al. 2013). In TtCsm6, the cleft is 30 Å long, 15 Å deep, and 13 Å wide at its narrowest point. Its base is formed by the interlocking of the α18–α19 loops provided by the two HEPN domain protomers. These loops contain the conserved R-X_4-6_-H motif, which has been shown to mediate ribonuclease activity in other HEPN domain proteins. Consistent with the observed ssRNA-selective ribonuclease activity of TtCsm6, the width of the cleft would permit binding of a single-stranded nucleic acid substrate, while sterically excluding double-stranded nucleic acids. Taken together, the structural features of TtCsm6 dimer thus suggest that the protein contains two ligand-binding sites: a putative allosteric ligand-binding site at the interface of the CARF domains, and an ssRNA-binding cleft located at the HEPN domain interface, which harbors the ribonuclease active center.

### The HEPN domain dimer forms a composite ribonuclease active site

**FIGURE 3.**
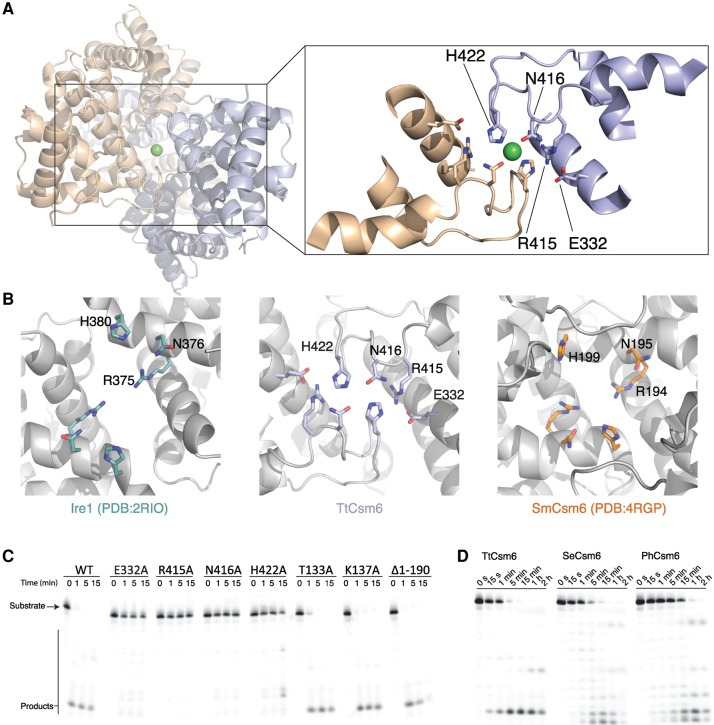
The C-terminal HEPN domains of TtCsm6 form a composite endoribonuclease active site. (*A*) Overall view of the HEPN domain interface in the TtCsm6 dimer. The *inset* shows a close-up view of the HEPN domain active site. Conserved active site residues are shown in stick format. The Ni^2+^ ion present in the TtCsm6 crystal structure is shown as a green sphere. (*B*) Comparison of the HEPN ribonuclease active sites of yeast Ire1 (*left*), TtCsm6 (*middle*), and SmCsm6 (*right*). Active site residues are shown in stick format. The structures were aligned by least-squares superposition of the active site Asn, Arg, and His residues and are shown in identical orientations. (*C*) Nuclease activity assays of active site mutants of TtCsm6. The assays were performed using a 24-nt ssRNA substrate labeled with Cy5 at the 3′-end. Reactions were resolved on a 16% denaturing polyacrylamide gel and analyzed on a fluorescence gel scanner. For conciseness, the panel depicts a cropped region of the denaturing gel; a full-size image of the gel is shown in Supplemental Figure 7. (*D*) Ribonuclease activities of Csm6 orthologs. Recombinant *T. thermophilus*, *S. epidermidis* (SeCsm6), and *P. horikoshii* (PhCsm6) Csm6 proteins were incubated with a 24-nt Cy5-labeled ssRNA substrate at 37°C. Reactions were sampled at indicated time points and analyzed as for Figure 1C. For conciseness, the panel depicts a cropped region of the denaturing gel; a full-size image of the gel is shown in Supplemental Figure 8.

Site-directed mutagenesis studies have shown that the conserved R-X_4-6_-H motif is responsible for the catalytic activity in a number of dimeric HEPN domain-containing ribonucleases, including tRNA anticodon nucleases PrrC and RloC, as well as the eukaryotic pseudokinase–ribonuclease enzymes Ire1 and RNase L (Davidov and Kaufmann 2008; Lee et al. 2008; Meineke and Shuman 2012; Han et al. 2014; Huang et al. 2014). The molecular architecture of the TtCsm6 HEPN domain dimer is highly similar to that of the kinase-ribonuclease Ire1 (Supplemental Fig. 6A,B). In the TtCsm6 structure, the floor of the HEPN domain cleft is lined with side chains of the invariant residues Arg415, Asn416, and His422 from the R-X_4-6_-H motif. The Asn416/His422 side chain pairs from the two HEPN domains bind a single Ni^2+^ ion in a tetragonal planar coordination (Fig. 3A). As TtCsm6 and other HEPN domain ribonucleases are divalent metal-independent enzymes (Fig. 1B; Anantharaman et al. 2013), the observed Ni^2+^ ion binding in the putative active site of TtCsm6 might be an artefact due to crystallization in the presence of nickel(II) chloride. In SmCsm6, the R-X_4-6_-H motif residues reside in an α helix, whereas the TtCsm6 R-X_4-6_-H motif adopts an irregular loop conformation, suggesting that the TtCsm6 active center might be distorted as a result of Ni^2+^ binding (Fig. 3B). Nevertheless, the disposition of the putative active site residues in TtCsm6 broadly resembles that observed in Ire1 and the related RNase L (Fig. 3B; Supplemental Fig. 6), suggesting that Csm6 proteins use a similar mechanism of RNA hydrolysis (Lee et al. 2008; Han et al. 2014; Huang et al. 2014). To confirm the catalytic function of the putative active site residues in TtCsm6, we probed the ribonuclease activity of TtCsm6 proteins in which the invariant R-X_4-6_-H motif residues (Arg 415, Asn416, and His422) as well as a nearby glutamate (Glu332) were substituted by alanine. As a control, we tested TtCsm6 proteins in which the conserved CARF domain residues Thr133 and Lys137 were mutated. All mutant proteins were dimeric, as judged by size-exclusion chromatography (Supplemental Fig. 3). Individual substitutions of the HEPN domain residues led to near-complete loss of ribonuclease activity, whereas mutations in the CARF domain had little effect (Fig. 3C; Supplemental Fig. 7). This indicates that the ribonuclease activity of TtCsm6 is dependent on the presence of an intact R-X_4-6_-H motif in the HEPN domain while the CARF domain (D/N)-X-(S/T)-X_3_-(R/K) motif is not directly involved in RNA hydrolysis. Additionally, to test whether the HEPN domain is sufficient for ribonuclease activity in TtCsm6, we generated a truncated TtCsm6 protein lacking the N-terminal CARF domain (Δ1-190). This protein was as active as full-length TtCsm6, showing that the CARF domain is not required for enzymatic activation (Fig. 3C; Supplemental Fig. 7). Collectively, these results confirm that the ribonuclease activity of TtCsm6 is mediated by the R-X_4-6_-H motif in the HEPN domain independently of the CARF domain. In an analogy with the catalytic mechanisms of Ire1 and RNase L (Lee et al. 2008; Han et al. 2014; Huang et al. 2014), we propose that the dimerization of the HEPN domains and the resulting juxtaposition of the R-X_4-6_-H motifs lead to the formation of a composite symmetric active center that binds the substrate RNA asymmetrically (Supplemental Fig. 6B). The observation that TtCsm6 is a metal-independent ribonuclease that generates products containing a free 5′-hydroxyl group suggests that RNA hydrolysis involves a nucleophilic attack by the 2′-hydroxyl group to yield a 2′,3′ cyclic phosphate, as shown for other HEPN domain ribonucleases such as Ire1 and PrrC (Amitsur et al. 1987; Gonzalez et al. 1999), and implies that the HEPN active site residues in Csm6 likely serve the same functions as their counterparts in Ire1 (Lee et al. 2008). In the putative catalytic mechanism of Csm6, the active site histidine His422 in TtCsm6 would thus function in general acid–base catalysis, deprotonating the attacking 2′-hydroxyl nucleophile and/or protonating the leaving 5′-hydroxyl group, while Arg415 and Asn416 might mediate substrate binding and transition state stabilization through ionic and hydrogen bonding contacts.

### Ribonuclease activity is conserved in Csm6 orthologs

Our structural and biochemical analysis of TtCsm6 suggests that most Csm6 proteins are active ribonucleases. While the biological function of TtCsm6 has not been studied in vivo, deletion of the Csm6-encoding gene in *S. epidermidis* impairs anti-plasmid immunity mediated by the type III-A CRISPR–Cas system (Hatoum-Aslan et al. 2014). We therefore examined *S. epidermidis* Csm6 (SeCsm6) as well as Csm6 from *Pyrococcus horikoshii* (PhCsm6), a hyperthermophilic euryarchaeon, to probe whether these orthologs also possess ribonuclease activities. Both proteins contain the canonical R-X_4-6_-H HEPN domain motif and were therefore predicted to be enzymatically active. We heterologously expressed and purified Csm6 proteins and assayed their activities alongside TtCsm6. Both SeCsm6 and PhCsm6 proteins were able to degrade a 24-nt ssRNA substrate at 37°C, confirming that they are active ribonuclease enzymes (Fig. 3D; Supplemental Fig. 8). The enzymes produced overlapping, but nonidentical product patterns, suggesting that they may have slightly different substrate specificities. Together, these observations suggest that endoribonuclease activity is a general feature of Csm6 proteins. Furthermore, the conservation of the ribonuclease activity across different prokaryotic phyla strongly hints at its functional significance in the context of type III-A CRISPR-Cas systems.

### Conclusions

Type III-A CRISPR–Cas systems have recently been shown to target both ssRNA as well as dsDNA in a crRNA-guided manner (Tamulaitis et al. 2014; Samai et al. 2015). The enzymatic activities associated with these interference mechanisms reside within the Csm interference complex. The Csm3 subunits of the Csm complex catalyze ssRNA degradation. In turn, cleavage of a dsDNA target requires active transcription across the target site and is dependent on the Csm1/Cas10 subunit. The dual targeting specificity of the Csm complex is distinct from those of type I and II CRISPR-Cas systems. In addition to the Csm complex, a number of type III-A CRISPR–Cas systems include the HEPN domain-containing protein Csm6 that appears to be essential for their function (Hatoum-Aslan et al. 2014). Based on the presence of a conserved R-X_4-6_-H motif in their HEPN domains, Csm6 and Csx1 proteins were predicted to posses ribonuclease activities (Anantharaman et al. 2013). Consistently, we show here that Csm6 proteins from three divergent prokaryotes are active RNases in vitro and that the R-X_4-6_-H motif is required for ribonuclease activity in TtCsm6. The structure of TtCsm6 reveals that dimerization of the HEPN domains brings the R-X_4-6_-H motifs of the two HEPN domain protomers into close proximity, generating a composite ribonuclease active site that resembles those found in KEN domain ribonucleases Ire1 and RNase L (Lee et al. 2008; Han et al. 2014; Huang et al. 2014). Together, our structural and biochemical studies demonstrate that a subset of type III-A CRISPR–Cas systems contain an additional ribonuclease module that may contribute to the interference mechanisms of these systems and further expand their capabilities against a broad spectrum of nucleic acid invaders.

Although Csm6 is required for efficient anti-plasmid interference mediated by the *S. epidermidis* type III-A CRISPR–Cas system (Hatoum-Aslan et al. 2014), the functional importance of the ribonuclease activity in Csm6 (and the related Csx1 proteins) is presently unclear. It is possible that these proteins degrade RNA transcripts of DNA invaders, thereby augmenting the DNA- and RNA-targeting endonuclease activities of the Csm complex. Notably, the RNA cleavage activity of the Csm complex itself is not essential for efficient immunity against DNA plasmids in this context (Samai et al. 2015). An intriguing alternative is that Csm6/Csx1 proteins contribute to CRISPR immunity by targeting host (i.e., self) transcripts in order to induce dormancy or promote programmed cell death of the host. In this way, these proteins could provide a backup mechanism to restrict propagation of the nucleic acid invader if the endonuclease activities of the Csm effector complex are insufficient, as recently proposed (Makarova et al. 2012). The presence of a highly conserved pocket located at the dimer interface of the CARF domains in Csm6 proteins additionally suggests that their catalytic activity might be regulated in a ligand-dependent manner. The crystal structure of TtCsm6 reveals that its dimeric architecture would be highly suited for allosteric control, as conformational changes induced in the CARF domain dimer by ligand binding could be transmitted by the 6H domain to the ribonuclease active center at the HEPN domain interface, thereby regulating its substrate binding affinity or catalytic activity. Ligand-dependent control of RNA degradation by Csm6 could thus provide yet another layer of host genome defense in type III CRISPR-Cas systems.

## MATERIALS AND METHODS

### Cloning, expression and purification

The sequences encoding TtCsm6 (TTHB152), PhCsm6 (PH_RS00765), and SeCsm6 (SERP_RS12035) were PCR-amplified from their respective genomic DNAs and inserted into a pET-based expression vector (2HRT, Addgene ID 29718) using ligation-independent cloning. The resulting fusion protein constructs contained an N-terminal hexahistidine tag followed by a tobacco etch virus (TEV) protease cleavage site and the full-length protein sequence. Point mutations were introduced using inverse PCR and verified by DNA sequencing. All proteins were expressed in *Escherichia coli* BL21 Rosetta 2 (DE3) cells. Cultures were grown at 37°C in the presence of 100 µg/mL ampicillin and 33 μg/mL chloramphenicol to an optical density (OD_600nm_) of ∼0.7. Expression was induced by adding IPTG to a final concentration of 200 µM and the cultures were grown at 18°C for 16 h. Harvested cells were lysed by sonication in lysis buffer containing 20 mM HEPES pH 8.0, 500 mM KCl, 5 mM imidazole, supplemented with protease inhibitors. The clarified lysate was applied to a 10-mL HIS-Select Nickel Affinity Gel (Sigma) column and the column was subsequently washed with five column volumes of the same buffer. Bound proteins were eluted with lysis buffer supplemented with 250 mM imidazole. TEV protease was added to the eluted fraction and the sample was dialyzed against 20 mM HEPES 7.5, 500 mM KCl at 4°C for 16 h. To remove the hexahistidine tag and protein contaminants, the dialyzed proteins were re-applied to a HIS-Select Nickel Affinity Gel column. The flow-through fraction was collected, concentrated and further purified by size exclusion chromatography using a Superdex 200 (26/600) column (GE Healthcare Life Sciences) in 20 mM HEPES 7.5, 500 mM KCl. Eluted Csm6 proteins were concentrated to 15–50 mg mL^−1^.

### Crystallization and structure determination

Purified TtCsm6 protein was crystallized at 20°C using the hanging drop vapor diffusion method by mixing equal volumes of protein and reservoir solution. Initial crystals were obtained in 0.1 M HEPES pH 7.5, 12% (w/v) PEG 3350, 5 mM CdCl_2_, 5 mM CoCl_2_, 5 mM NiCl_2_, and 5 mM MgCl_2_ at protein concentrations ranging from 6 to 25 mg mL^−1^. The optimal crystal growth condition was subsequently refined to 0.1 M HEPES pH 7.5, 14% (w/v) PEG 3400 and 35 mM NiCl_2_ at protein concentrations ranging from 5 to 10 mg mL^−1^. Iterative rounds of microseeding yielded large single crystals that typically formed within few days and were fully grown within 1–2 wk. For cryoprotection, crystals were transferred into 0.1 M HEPES pH 7.5, 12% (w/v) PEG 3400, 35 mM NiCl_2_ and 35% (v/v) ethylene glycol and were flash-cooled in liquid nitrogen. The crystals belonged to space group *P*2_1_2_1_2 and contained two TtCsm6 molecules in the asymmetric unit. X-ray diffraction data were measured at beamline X06DA (PXIII) of the Swiss Light Source (Paul Scherrer Institute, Villigen, Switzerland). The data were indexed, integrated and scaled in XDS (Kabsch 2010). Native data were measured to a resolution of 2.30 Å. Experimental phases were obtained from a single-wavelength anomalous diffraction experiment measured at the Ni K-edge wavelength (1.48502 Å). Anomalous scatterers were located using Phenix.hyss (Zwart et al. 2008). Phasing and density modification were carried out in Phenix.autosol (Terwilliger et al. 2009), resulting in a readily interpretable electron density. The atomic model was built manually in Coot and refined using Phenix.refine (Afonine et al. 2012). The final model contains residues 1–167, 170–345, 349–367, and 371–464 in TtCsm6 molecule A, residues 1–15, 22–164, 171–346, 349–367, and 371–464 in molecule B, 76 water molecules and 6 Ni^2+^ ions.

### Nuclease assays

All synthetic RNA and DNA oligonucleotides were purchased from Microsynth AG unless stated otherwise. Cleavage assays to determine substrate specificity were performed with 24 nt 3′-end Cy5 labeled synthetic RNA1 (ACUGCAACGCAAUAUACCAUAGCU) and its nonlabeled complementary strand RNA2 (AGCUAUGGUAUAUUGCGUUGCAGU). Corresponding DNA substrates were 3′-end Cy5 labeled DNA1 (ACTGCAACGCAATATACCATAGCT) and its nonlabeled complementary strand DNA2 (AGCTATGGTATATTGCGTTGCAGT). DNA, RNA, and proteins were quantified with a NanoDrop spectrophotometer using the calculated extinction coefficients at 260 nm for RNA1 (230 900 M^−1^ cm^−1^), RNA2 (223 700 M^−1^ cm^−1^), DNA1 (236 100 M^−1^ cm^−1^) and DNA2 (234 100 M^−1^ cm^−1^) and at 280 nm for TtCsm6 (105 660 M^−1^ cm^−1^ for homodimer), PhCsm6 (109 780 M^−1^ cm^−1^) and SeCsm6 (107 080 M^−1^ cm^−1^). To determine TtCsm6 substrate specificity 200 nM RNA1 were incubated with 400 nM TtCsm6 homodimer in 20 mM HEPES pH 7.5 and 50 mM KCl and the reaction was incubated for 1 h at 37°C. To prepare dsRNA and dsDNA substrates, RNA1 and RNA2 as well as DNA1 and DNA2 oligonucleotides were preincubated in a 1:2 stoichiometry, heated to 75°C and subsequently slow-cooled to room temperature. Cleavage reactions using dsRNA, ssDNA, and dsDNA were set up the same way as for ssRNA. All reactions were quenched by addition of 0.5 volumes formamide supplemented with 0.005% (w/v) bromophenol blue. Twenty microliters of the sample were loaded on a 15% denaturing (7 M urea) polyacrylamide gel and resolved in 0.5× TBE at 55 W for 3 h. The gels were visualized using a Typhoon FLA 9000 laser gel scanner (GE Healthcare).

Nuclease activity assays to confirm endonuclease activity were performed as follows. One hundred nanomolar RNA1 or RNA3 (identical oligonucleotides that carry a Cy5 label on the 3′-end and 5′-end, respectively) were mixed with 200 nM TtCsm6 homodimer in 20 mM HEPES pH 7.5 and 50 mM KCl (in a total reaction volume of 150 µL) and the reactions were incubated at 37°C. Twenty microliter samples were taken at indicated time points and reactions were quenched by addition of 0.5 volumes formamide supplemented with 0.005% (w/v) bromophenol blue. An RNase T1 control digest was performed under the same conditions by substituting TtCsm6 with 0.06 U of RNase T1 (Thermo Scientific). The samples were resolved and visualized as above.

Ribonuclease activity assays to determine substrate preference of TtCsm6 were conducted with synthetic homo-oligomeric RNAs (A_12_, C_12_, and U_12_, respectively, obtained from Integrated DNA Technologies) labeled with Cy5 at their 3′ termini. Two-micromolar substrates were incubated with 8 µM TtCsm6 at 37°C in 20 mM HEPES pH 7.5 and 50 mM KCl in a total volume of 25 µL. Three-microliter samples were taken at indicated time points and the reactions were quenched by addition of 1 volume formamide supplemented with 0.005% bromophenol blue. The samples were resolved on a 20% denaturing (7 M urea) polyacrylamide gel and visualized as above.

In assays to determine the chemistry of RNA cleavage by TtCsm6, 4.5 µM RNA1 was mixed with 20 µM TtCsm6 in 2 mM HEPES pH 7.5 and 50 mM KCl in a total reaction volume of 15 µL. The reaction was incubated at 37°C for 15 min, stopped by heat inactivation at 95°C for 15 min and centrifuged to remove precipitated denatured TtCsm6 protein. The supernatant was equally split into three 4-µL aliquots. The first sample remained untreated. The second sample was mixed with 1 mM ATP and 1× T4 Polynucleotide Kinase buffer in a total volume of 10 µL (Thermo Fisher Scientific, final concentrations). The third sample was treated the same way, but 0.5 U/µL T4 Polynucleotide Kinase were added to the reaction (Thermo Fisher Scientific). All samples were then incubated at 37°C for 1 h and subsequently mixed with 0.5 volumes of formamide supplemented with 0.005% bromophenol blue. The alkaline hydrolysis ladder was generated as follows. 2.5 µM RNA1 was incubated with 150 mM NaHCO_3_, pH 9.6, at 95°C for 1.5 h. Hydrolysis was stopped by addition of 150 mM HCl and the sample was mixed with 0.5 volumes of formamide supplemented with 0.005% bromophenol blue. All samples were resolved on a 20% denaturing (7 M urea) polyacrylamide gel at 25 W for 4.5 h.

To perform TtCsm6 mutant cleavage assays, 1 µM RNA1 was incubated with 2 µM TtCsm6 homodimer in 20 mM HEPES, pH 7.5, and 50 mM KCl at 37°C (in a total reaction volume of 50 µL). Ten-microliter samples were taken at indicated time points and reactions were quenched by addition of 0.5 volumes formamide supplemented with 0.005% (w/v) bromophenol blue. The samples were resolved on a 16% denaturing (7 M urea) polyacrylamide gel and visualized as above.

Nuclease activity assays of Csm6 orthologs were performed by mixing 250 nM RNA1 with 500 nM TtCsm6, 500 nM SeCsm6 or 5 µM PhCsm6 in 20 mM HEPES, pH 7.5, and 50 mM KCl in a total reaction volume of 80 µL. Reactions were then incubated at 37°C. Ten-microliter samples were taken at indicated time points and quenched by adding 0.5 volumes of formamide supplemented with 0.005% bromophenol blue. The samples were resolved on a 20% denaturing (7 M urea) polyacrylamide gel and visualized as above.

## DATA DEPOSITION

Atomic coordinates and structure factors of TtCsm6 have been deposited in the Protein Data Bank under PDB code 5FSH.

## SUPPLEMENTAL MATERIAL

Supplemental material is available for this article.

## Supplementary Material

Supplemental Material

## NOTE ADDED IN PROOF

While this manuscript was in revision, another study reported that the *Pyrococcus furiosus* Csx1 protein is an adenosine-specific ribonuclease (Sheppard et al. 2016). These results are in broad agreement with our structural and biochemical data on TtCsm6.
