# Supplementary material for: Structural basis for the endoribonuclease activity of the type III-A CRISPR-associated protein Csm6
Source: RNA. 2016 Mar;22(3):318–29. doi: 10.1261/rna.054098.115 (PMC4748810; doi:10.1261/rna.054098.115)
Supplement: Supplemental Material [file supp_22_3_318__index.html]

Structural basis for the endoribonuclease activity of the type III-A CRISPR-associated protein Csm6 — Structural basis for the endoribonuclease activity of the type III-A CRISPR-associated protein Csm6 — Supplemental Material 

# Structural basis for the endoribonuclease activity of the type III-A CRISPR-associated protein Csm6

## Supplemental Material

**Files in this Data Supplement:**

- Supp Data File 1.pdf
- Supp Material.pdf
