# Supplementary material for: Structural basis for the endoribonuclease activity of the type III-A CRISPR-associated protein Csm6
Source: RNA. 2016 Mar;22(3):318–29. doi: 10.1261/rna.054098.115 (PMC4748810; doi:10.1261/rna.054098.115)
Supplement: Supplemental Material [file supp_054098.115_SuppDataFile1.pdf]

Supplemental Data File 1. Multiple sequence alignment of TtCsm6 homologs.
50 closest homologs of TtCsm6 (gi|499548365) were identified by Psi-BLAST and aligned using COBALT.

```
gi|499548365                  ME---------D----LDALWERYREAVRAGGNPQA-------LYQEMV----WPALLAL
gi|570755047                  ME---------D----LDALWERYREAVRAGGNPQA-------LYQEMV----WPALLAL
gi|499487817                  ME---------D----LDALWERYREAVRAGGNPQA-------LYQEMV----WPALLAL
gi|551068805                  MAENPLDRAGGD----LEALWLQYKEAVRAGGNPHD-------LYQKMV----WPTLLEK
gi|516806813                  MHD-------EG----LENVWQAYKEAVRAGGNPQT-------LYQEQV----WPALLDL
gi|740203173                  ME--PLPNQGLD----LEALWQSYKEAVNAGGNAQA-------LYQERI----WPVLLEK
gi|489138195                  MEE-------------LQALWDRYKEAVRSGGNPQA-------LYQEMV----WPALVAR
gi|518381449                  MQD-------------LATLWEEYKQAVRAGG-----------DAVGLYRERVWPALLER
gi|746416481                  MQD-------------LATLWEEYKQAVRAGG-----------DAVGLYRERVWPALLER
gi|503470028                  MPEKALNDPQAL-KEALEAAWAEYKARVEEGG-----------DPQTLYRERVWPLLLAL
gi|740224592                  MQT--VNNPGD-----LEPIWQAYREAVRAGG-----------NPHTLYREMVWPHLLAL
gi|746419752                  MLQ--DQERWNQ----LQEAWARYKEAVGAGGNPQA-------LYQEMV----WPHLLEL
gi|740218035                  MNER------------LGKLWQEYKEKVKAGQ-----------DPRTLYQQMVWPILLDS
gi|517272690                  M--P------SL-QAQLEEARKTLYERLDRGE-----------DPTPFYNEAVWPLLLAL
gi|511098904                  MGAE------SL-RNRLEEARKTLYERLDRGE-----------DPTPFYNEAVWPLLLAL
gi|753938889                  -------------MQKIHLAWQHLKTQELEGA-----------RAQELYNQTVWPLLLEL
gi|296849607                  MED--------L-MQKIHLAWQHLKTQELEGA-----------RAQELYNQTVWPLLLEL
gi|701167105                  MENH------PL-EEILERKWEEYKTKVKEGG-----------NAQKIYQEEVWPILLQL
gi|502941971                  MAEV--VEP-----VELNDLWQQFKEMHASQGL---------EAANDFYDQHVWPSLLLR
gi|648543053                  MNRN---------KERLRQLWTRYKDLLGQGNIGRTEPSPQAKEARELYEKEIWPLT---
gi|654417026                  MNQN---------KERLRELWAEYKTLIRQESADRAGPAPQGQRAKELYDTQIWPLT---
gi|502777546                  MNQN---------KERLRELWAEYKTLIRQESANRAGSSPQSQKAKELYDTQIWPLT---
gi|654402559                  MNQN---------KERLRELWVQYKTLIRQERANRGESSPKTPTAKDLYDTQIWPLT---
gi|302777911                  MKEE-LKLTEEE----IERLLKEKTEIWETMQAKNA---------EEYYYNEIFPLVVDK
gi|779961749                  MKEE-LKLSNDK----IESLLNEKAKIWETMQAKRA---------EEYYYDEIFPLVVNK
gi|505124139                  MKEE-LKLSNDK----IESLLNEKAKIWETMQAKRA---------EEYYYDEIFPLVVNK
gi|502759809                  MESE-GILSNSE----IEKKIKEKVEIWETKPAKEA---------EDYYYQEIFPLVVKK
gi|655943915                  MESE-GILSNSE----IEKKIKEKVEIWETKPAKEA---------EDYYYQEIFPLVVKK
gi|125715920                  ------------------------------------------------------------
gi|489607383                  MSDI-----YAR----MKEKRDIWYAMDRSNNEERM-------MAEEYYKKELMPLIIEY
gi|754095284                  -------------------------------------------------------LIIEY
gi|489611620                  M-EL-----YKD----LMKKTIFWREMDRSSEKLRI-------ASEEYYKKELMPLIIEY
gi|504021345                  MNDI-----YIK----MKEKRDMWYAMDRSNNERRI-------LAEQYYKKELMPLIIEY
gi|653611173                  M-EL-----YKD----LMKKTIFWREMDRSSEKLRI-------ASEEYYKKELMPLIIEY
gi|605572971                  MKTF-----KES----FVQATAQWKLLNRGTEEERR-------QADAFYKDHILPEVMMH
gi|740811186                  MKTF-----KES----FVQATAQWKLLNRGTEEERR-------QADAFYKDHILPEVMMH
gi|516314532                  MDRT-------A----LDQVIEQWQSLNNSEERSQ------------FYDNHVFPALKE-
gi|655011063                  MD--------------FSEQLQRFRERVGNRRAE--------------------------
gi|670513542                  MD--------------FSTQLQQFRERVGNRQAV--------------------------
gi|500595682                  M---------------------------TASDKRI-------PFPEIF-----------
gi|301057067                  MLN----TSDHGPVVDFGACVDRYHDIHQGRQGYGDGTRA--EQATRFYLDHLLGETATR
gi|757183905                  MNKELEKR------------TQEWMGLERATLKQRT-------KAEEYYEKQLMKLIVDS
gi|501216200                  MD--------------FSEQLERFIARVDKRRTP--------------------------
gi|501070122                  M---------------------------AQPTEAL-------PFPDMF-----------
gi|703489217                  MTG----EEAH-----FDELAAQMRRISLGEESYGDGLRPH-EQANEFYLDRLLGESVAR
gi|559063203                  MGSL---DEKH----------SRMRRIFRGEEEYGGGTAA--EQAQAFRLAEIYDEAVEV
gi|490153543                  MNKELSR------------LTEQWKQMERKTEQQRR-------LADAFYETDMMRLIEED
gi|548231998                  MADETKRKTADETENPLQMMTEQWMQLERKTTKQRE-------AADLFYEKKLMNLIEDN
gi|656268823                  MTVI---GSSRA----FADELAAFRARTRDLGFR--------------------------
gi|497885042                  MKHH------------LAVYYEKLRQ-----NKDEA---------KEYYFQHMFDNVCHE
gi|585249330                  ------------------------------------------------------------


gi|499548365                  WRE---KPRVYPFPQAFAVSVHTLGT-SPEATALAILGAGAERVYVLHTPESARF--LPR
gi|570755047                  WRE---KPRVYPFPQAFAVSVHTLGT-SPEATALAILGAGAERVYVLHTPESARF--LPR
gi|499487817                  WRE---KPRVYPFPQAFAVSVHTLGT-SPEATALAILGAGAERVYVLHTPESARF--LPR
gi|551068805                  WRK---EPSVYPSPQPFAVSIHTLGT-SPEATALAILGGGAEQVYVLHTAESARF--LPR
gi|516806813                  WRR---QPRVYPSRQPFAVSVHTLGT-SPEATALAILGTGAETVYVIHTRDSASF--LER
gi|740203173                  WRQ---DPPVYPSRQPFQVSIHTLGT-SPEATALAILGAGAGDVYVLHTPESARF--LSR
gi|489138195                  WKE---APNVHPRREAFAVSVHTLGT-SPEATILAILGAGAERVYVLHTRESASY--LER
gi|518381449                  WRQ---EPPVHPSPQEFAVSIHTLGT-SPEATVLAILGTRAREVYVLHTDLTRAH--VPR
gi|746416481                  WRQ---EPPVHPSPQEFAVSIHTLGT-SPEATVLAILGTRAREVYVLHTDLTRAH--VSR
gi|503470028                  WRA---EPPVHPGRQTFALSIHTLGT-SPEAAIQAILGTGAEEVYVFHTEETRRF--LPR
gi|740224592                  WRT---APQVYPERRSFRVSIHTLGT-SPEATTLAVLGTGAEEVYVLHTEETRRH--LDQ
gi|746419752                  WRH---APPVSPERMVFRASLHTLGT-SPEATALAILGTGAQEVYVLHTEESRRF--LER
gi|740218035                  WKK---SPVVEPQKFQFDVSIHTLGT-SPEATTLAILGTRSSEVYVIHTPETRKY--LEQ
gi|517272690                  WRE---DPPVRPRFQPYEVSIHTVGT-SPEATILAILGTGAERVYLLHTEESQRY--LER
gi|511098904                  WRE---DPPVRPRFQPYEVSIHTVGT-SPEATILAILGTGAGRVYLLHTEESQRY--LER
gi|753938889                  WRL---EPQVHPLRESFDVSIHTLGT-SPEATTLAALGLGADEIYVLHTPESRRY--LAQ
gi|296849607                  WRL---EPQVHPLRESFDVSIHTLGT-SPEATTLAALGLGADEIYVLHTPESRRY--LAQ
gi|701167105                  WKE---RPKVIPAPKKFDISIHTLGT-SPEATILAILGTQADEIYILHTPETKAF--IAK
gi|502941971                  WRA---QD----HGPRCNLSLHTLGT-SPEALILAATALRSEQIYIFYTDESEKY--LDR
gi|648543053                  --K---EGFVDRGQQQYKASFHTVGT-TPEPVILSARALNAEKVYLLHTKDTEKL--CER
gi|654417026                  --K---EGFTDRGQQRYLASFHTVGT-TAEPVILSVRALDADKVYLLHTKDTEKV--CGR
gi|502777546                  --K---EGFTDRGQQQYVASFHTVGT-TAEPIILSVRALDAEKVYLLHTKDTEKL--CGR
```

```
gi|654402559                    --K---EGFTDRGQRQYVASFHTVGT-TIEPVVLSTRALDAEKVYLLHTKDTERL--CGQ
gi|302777911                    FRL---SISDEIQGK-YENLILSLGM-SFEPLVLTITALKPKRVLFLYTEDSLKN--LDE
gi|779961749                    FHL---SINNEFYGK-YENLILSLGM-SFEPLVLTIKALKPKRVLFLYTEDSLKN--LDE
gi|505124139                    FHL---SINNEFYGK-YENLILSLGM-SFEPLVLTIKALKPKMVLFLYTEDSLKN--LDE
gi|502759809                    FHL---ETDVSFKEK-YENLILSLGF-SFEPLVLTISFIKPKQVLFLYTKDSREN--LDK
gi|655943915                    FHL---ETDVSFKEK-YENLILSLGF-SFEPLVLTISFIKPKQVLFLYTKDSREN--LDK
gi|125715920                    ------------------MVL-TLGT-SYEPLVLSISVLKPEKVLILYTDKSHHL--LDD
gi|489607383                    FIQ---ENTKKISGNCDAMVL-TLGT-SYEPLVLSISVLKPEKVLILYTDKSHHL--LDD
gi|754095284                    FIQ---ENTKKISGNCDAMVL-TLGT-SYEPLVLSISVLKPEKVLILYTDKSHHL--LDD
gi|489611620                    FIQ---ENAKKISGSCDAMVL-TLGT-SYEPLVLSISVLKPEKVLILYTDKSHHL--LDD
gi|504021345                    FIQ---ENAKKVTGNCDAMVL-TLGT-SYEPLVLSIAVLKPEKVLILYTDKSHHL--LDE
gi|653611173                    FIQ---ENAKKISGSCDAMVL-TLGT-SYEPLVLSIAVLKPEKVLILYTDKSHHL--LDD
gi|605572971                    FRE---K---YAHSESCENLILTLGT-SYEPLVLSILALRPERVLIMHSTRTRPL--LDD
gi|740811186                    FRE---K---YAHSESCENLILTLGT-SYEPLVLSILALRPERVLIMHSTRTRPL--LDD
gi|516314532                    YVI---PREQQKHPQAYDCLILSVGL-SPEPIILSVLTFRPTQVCLLYTAESKKL--LDR
gi|655011063                    -------------------ALILAGSRQEDTAALLIGALQPNRVAFLLTPETYDF--LRK
gi|670513542                    -------------------ALILAGSRQEDTAALLIGALQPKRVAFLLTPETHDF--LEK
gi|500595682                    --A---SFRATADGCAFRGLV-LVGTLQADTPALLIAGLNPERVAFLLTDQSRPK--LDE
gi|301057067                    -AR---AESRLPARP-VDLLISLCGF-TPVPTVLTHELLRPKRMVVVVSRDAEDS--IDV
gi|757183905                    FSE---NNKESIVGKT-DYLILSVGT-SYEPLVLSISLLNPKKIMFLYTEKSEAS--IDK
gi|501216200                    -------------------ALIIPGSRQADTAALLIAALRPERVAFLLTPETTNF--PAQ
gi|501070122                    --Q---RFRSTIGDHAFRGLV-LVGTLQADTPSLLIAGLNPERVAFLLTDQSREKGLVAQ
gi|703489217                    -AS---RSLAQPPGD-VDLLISLCGF-SPTTTILAFELLKPRRLVVITSEDAEES--INV
gi|559063203                    -AQ---ANSADVERPEVDLLISLSGF-SPETTLLAFALTRPTRILIITSEGTQKT--IDT
gi|490153543                    FIA---RNREQVFEQA-DYFIVSVGT-SYEPIVLNMKLFQPDRILFLYTEKTEDV--LYK
gi|548231998                    FIA---RNRERVEEEV-EYLIMSVGT-SYEPLVLSIKLLNPQKILFLCTAITEKY--LDK
gi|656268823                    -------------------GLVLLGSRQSTTAALLIGALQVERVAFLLTDETRQM--PDD
gi|497885042                    FKKKVAEKQANREIQEYDYLIMTVGY-NIEPLVMWIKALSPKRVFFLCSEDTEKY--VDE
gi|585249330                    ----------------------MSGL-TPLPTILAYDVLRPKHLLVIYSEKAAGA--VDV
                                                        *

gi|499548365                    LRQDTGKDLYP------------VEIGKSDVEAIYR-EVKRL-LEKHP-----EVP----
gi|570755047                    LRQDTGKDLYP------------VEIGKSDVEAIYR-EVKRV-LEKHP-----EVP----
gi|499487817                    LRQDTGKDLYP------------VEIGKSDVEAIYR-EVKRL-LEKHP-----EVP----
gi|551068805                    LREDTGKDLYP------------LEIGKSDVAAIYR-EVKRL-LERYP-----DVP----
gi|516806813                    LRRDTGKDLYP------------LEVGKSDVAAIYR-HVKDL-LERHP-----QVP----
gi|740203173                    LRQDTGKDLYP------------IEIGKSDVAAIYR-EVKRL-LEKHP-----GLP----
gi|489138195                    LQKETGKPIYP------------LEVGKSDVAAIYR-EVKRI-LDQHG-----DVP----
gi|518381449                    LREETG---KE---------IYPLEVGKSDVAAIYR-HVRYL-LERFP-----DVP----
gi|746416481                    LREETG---KE---------IYPLEVGKSDVAAIYR-HVRYL-LERFP-----DVP----
gi|503470028                    LREETG---KE---------VYPLEIQKSDVAAIYR-KVREL-LEKRP-----EAL----
gi|740224592                    LEADAN---RR---------VYPKEVHKSDVAQIYQ-QVYEI-LKRLP----KDTP----
gi|746419752                    LRQDTGVMIYP------------IEIGKSDVTAIYR-EVRKL-LERYP-----EEP----
gi|740218035                    IRRDVG---VE---------LYPIEIEKNDVVTIYK-RIKEV-VERNI-----GKA----
gi|517272690                    IQRETG---RE---------AYPIRVDKSDVALIYK-TVAEL-LGRHR-----GAR----
gi|511098904                    IQRETG---RE---------AYPIRVDKSDVTLIYK-TVAEL-LGRHR-----GAR----
gi|753938889                    LQQDLG---RP---------VYPIEIDKSDVTRLYQ-VVGEQ-VRKHP-----GKK----
gi|296849607                    LQQDLG---RP---------VYPIEIDKSDVTRLYQ-VVGEQ-VRKHP-----GKK----
gi|701167105                    LENETG---KK---------TYPIEVGKSDVAAIYN-EVIKI-LEKNP-----DKW----
gi|502941971                    VEAELG---KR---------VWAYNVSKDDPTTIYQ-RFQDV-LQRYP-----HAD----
gi|648543053                    IEKELGWGVDR---------IKTLQVSRSDPEDIYR-QVRQQ-IDRLD----PKDP----
gi|654417026                    IERELGWGVER---------IKTLLVGRSDPEDIYR-QVRQK-VDEIP----PDAA----
gi|502777546                    IERELGWGVER---------IKTLLVGRSDPEDIYK-QVRQK-VDELP----PDAA----
gi|654402559                    IEKELGWGVER---------IKTLLVGRSDPEDIYK-QVRQK-VDELP----PDAA----
gi|302777911                    VIKWTNLLPSQ---------YTAEEIVKDDPVDIYR-VLKKVYVDRWG----CPDK----
gi|779961749                    VIKWTNLLPSQ---------YVAEEIVKDDPVDIYR-VLKKVYVDRWG----SPVK----
gi|505124139                    VIKWTNLLPSQ---------YVAEEIVKDDPVDIYR-VLKKVYVDRWG----SPGK----
gi|502759809                    VIQWAGLLPSQ---------YVAEEIEKDNPVDIYR-VLKKIYVEKWN----KPDK----
gi|655943915                    VIQWAGLLPSQ---------YVAEEIEKDNPVEIYR-VLKKIYVEKWN----KPDK----
gi|125715920                    VIEFTKLKPSQ---------YVATDVDAENPLQLYR-KIKDV-YEKWG----RPRN----
gi|489607383                    VIEFTKLKPSQ---------YVATDVDAENPLQLYR-KIKDV-YEKWG----RPRN----
gi|754095284                    VIEFTKLKPSQ---------YVATDVDAENPLQLYR-KIKDV-YEKWG----RPRN----
gi|489611620                    VIEFTKLKPSQ---------YVATDVDAENPLQLYR-KIKDV-YEKWG----RPRN----
gi|504021345                    VIEFTKLKPSQ---------YIATDVDAENPLQLYQ-KIKDI-YEKWG----RPKN----
gi|653611173                    VIEFTKLKPSQ---------YIATDVDAENPLQLYQ-KIKDI-YEKWG----RPKN----
gi|605572971                    VIELTGLKPSR---------YISRMVNSENPLKLYQ-VVKEV-YEEWD----RPGK----
gi|740811186                    VIELTGLKPSR---------YISRMVNSENPLKLYQ-VVKEV-YEEWD----RPGK----
gi|516314532                    IIDEANLKASQ---------IEMAEISETRLQNIYQ-KIREV-YEKWE----RPTR----
gi|655011063                    VAIKLGREPDQ--------RWISRTAHYTDVTQVYR-ELRAV-IEEWSDL--DRDQ----
gi|670513542                    VAAKLDREPDP--------TWIKRIAHYTDVNQVYR-ELRAV-IEEWCDL--DRDQ----
gi|500595682                    VRQRLAQVADQAPLRCSPDDWFCPDGDYSSVLRVYT-GLRTV-LDRWRDL--ERHE----
gi|301057067                    IHDRVTRPSGPL----RPRDFRHASCDPTDPLSIYR-IVKEE-LDRTPAGDGRP-Y----
gi|757183905                    IVKFCGLDASI---------YDKRKVHETNPLSVYQ-EIKRA-YLMWN----RPDK----
gi|501216200                    MAERLGRQPDA--------GWLCKTARYTDINQVYR-ELRAV-IEAWSDL--EREQ----
gi|501070122                    CRARLEQVADQVSLRCLPDAWFCPNGDHSNVLSVYT-GLRAV-LDRWNDL--ERHE----
gi|703489217                    LGERIVG-SGKL----QHKDFLPRRCVPTDPLGIYR-IVAAE-LEEHARRVGRPSL----
gi|559063203                    IWEKLAG---KI----KFSEARHVTCDPVDPTSIYDIVLKEV-RSLLAA--GRPPR----
gi|490153543                    IVKYCKLEPDS---------YEKNKVSERNPLDIYR-EIKRQ-YLEWD----KPKK----
gi|548231998                    IVKHCNLPVSA---------FQKCIVSETEPMDIYR-EIKKA-YIEWG----KPEK----
```

```
gi|656268823                  VAALLGCSP-Q--------AWLCPQGNHNTTRAVYQ-GLRQV-LEQWADL--DRAA----
gi|497885042                  ICKRAELIHTQ---------CDHEVIRKTNGTDVYE-MINKF-IEKNR----IMQENKLN
gi|585249330                  IGDHVVGPGQLKA-----SQFSHVPVDPTDPRIVYQ-VINEY-LARFN-----PADGR--
                                                                  *                         

gi|499548365                  -VALDLTSGTKAMSAGLAAAGFFFQRFYPKVRVVYVDNEDY-DPELRRP--RAGTEKLRI
gi|570755047                  -VALDLTSGTKAMSAGLAAAGFFFQRFYPKVRVVYVDNEDY-DPELRRP--RAGTEKLRI
gi|499487817                  -VALDLTSGTKAMSAGLAAAGFFFQRFYPKVRVVYVDNEDY-DPELRRP--RAGTEKLRI
gi|551068805                  -VALDLTSGTKAMSAGLAAAGFFFQRFFPKVWVVYVDNEDY-DPELRRP--RAGTERLVI
gi|516806813                  -VALDLTSGTKAMSAGLAAAGFFFQRFFPQVRVVYVDNEAY-DPELRRP--RAGTERLVI
gi|740203173                  -VALDLTSGTKAMSAGLAAAGFFFQRFYPEVRVVYVDNEVY-DPELRRP--KAGTERLII
gi|489138195                  -VALDLTSGTKAMSAGLAVAGFFFRRFYPKARVVYVDNEDY-DSELRRP--RAGTERLII
gi|518381449                  -VALDLTSGTKAMSAGLAAAGFFFQRFYPEVRVVYVDNDDY-DPELRRP--RAGTERLII
gi|746416481                  -VALDLTSGTKAMSAGLAAAGFFFQRFYPEVRVVYVDNDDY-DPELRRP--RAGTERLII
gi|503470028                  -VALDVTGGTKAMSAGLAAAGFFFQRFFPGVRVVYVDSGEY-DVKLRRP--RAGTERLIV
gi|740224592                  -VALDITSGTKAMSAGLAAVGFFFRRHYSNLQVVYVDNEEY-DQELRRP--KAGTERLMI
gi|746419752                  -VALDVTSGTKAMSAGLAAAGFFFRRFFPKVKVVYVDNEDY-DVALRRP--RAGSEKLIV
gi|740218035                  -VALDVTGGTKAMSAGMASGGFFFRRFFPNIRVVYVDNEEY-DEELRRP--VAGSEKLVI
gi|517272690                  -VALDLTSGTKAMTSGLAAAAFFLQRIHSEVQVVYVDNEGY-DPRLRRP--IPGTEYLVR
gi|511098904                  -VALDLTSGTKAMTSGLAAAAFFLQRIHPEVQVVYVDNEGY-DPRLRRP--IPGTEYLVR
gi|753938889                  -IALDLTSGTKAMSAGMAAAGYFLQRVHPSLRVAYVDNDAF-DVALRKP--VAGTEKLIL
gi|296849607                  -IALDLTSGTKAMSAGMAAAGYFLQRVHPSLRVAYVDNDAF-DVALRKP--VAGTEKLIL
gi|701167105                  -VALDLTSGTKAMSSGLGAAGFFFRRFFDKLRVVYVDNDLY-DVEVRRP--KAGSERMVI
gi|502941971                  -IAVDITSGTKPMVTGLGAAAFSAMTFGYKLRVFYVNGE-F-DPAARRP--RAGSEALIQ
gi|648543053                  -IAFDPTGGTKAMVAGLAMFAFSLAEEGRIAHVYYVDNEEY-DDQLRRP--VAGTEFLKR
gi|654417026                  -IAFDPTGGTKAMVAGLAMFAFSLAEEGRTAHVYYVDNEEY-DDELRRP--VAGTEFLKR
gi|502777546                  -IAFDPTGGTKAMVAGLAMFAFSLAEEGRTAHVYYVDNEEY-DDELRRP--VAGTEFLKR
gi|654402559                  -IAFDPTGGTKAMVAGLAMFAFSLAEEGRTAHVYYVDNEEY-DDELRRP--VAGTEFLKR
gi|302777911                  -TAIDFTGGTKSMSAGIAMAGAYF-----KIDLVYVAS-DY-NNKMRKP--KPGTEKLKF
gi|779961749                  -TAIDFTGGTKSMSAGIAMAGAYF-----KIDLIYVAS-EY-NSKMRKP--KPGTEELKF
gi|505124139                  -TAIDFTGGTKSMSAGIAMAGAYF-----KIDLIYVAS-EY-NSKMRKP--KPGTEELKF
gi|502759809                  -TAIDFTGGTKAMSAGMAMAGAYL-----KIDLIYVAS-EY-NNKMRKP--WPGTEKLKV
gi|655943915                  -TAIDFTGGTKAMSAGMAMAGAYL-----KIDLIYVAS-EY-NNKMRKP--WPGTEKLKV
gi|125715920                  -IYVDFTGGTKSMAAGCAMAGSAI-----GAKLIYIAG-NF-LTDLRKP--EPGSEKLCY
gi|489607383                  -IYVDFTGGTKSMAAGCAMAGSAI-----GAKLIYIAG-NF-LTDLRKP--EPGSEKLCY
gi|754095284                  -IYVDFTGGTKSMAAGCAMAGSAI-----GAKLIYIAG-NF-LTDLRKP--EPGSEKLCY
gi|489611620                  -IYVDFTGGTKSMAAGCAMAGSAI-----GAKLIYIAG-NF-LTDLRKP--EPGSEKLCY
gi|504021345                  -IYVDFTGGTKSMAAGCAMAGSAI-----GAKLIYVGG-NF-LPDLRKP--EPGSEKLCY
gi|653611173                  -IYVDFTGGTKSMAAGCAMAGSAI-----GAKLIYVGG-NF-LPDLRKP--EPGSEKLCY
gi|605572971                  -IYVDFTSGTKSMTAGCAMAGSVI-----NAKFVYIAS-HY-LADLRKP--EPGSETLSY
gi|740811186                  -IYVDFTSGTKSMTAGCAMAGSVI-----NAKFVYIAS-HY-LADLRKP--EPGSETLSY
gi|516314532                  -IAVDPTGGTKAMVAGCTLAGNLI-----GADLVYVSS-DY-NTILRKP--NPGSESLIL
gi|655011063                  -ILVDLTAGTKPMTVGLAKAAYVL-----GLGAIYIESDY----EQNQF--VPGSQRLID
gi|670513542                  -ILVDLTAGTKPMTVGLAKAAYVL-----GLGTIYIESDY----EQNQV--VPGSQRLID
gi|500595682                  -IAVDLTGGKSTMTVGLAKAAHVL-----RLAAVYVDS----DYADGRP--VPGTQRLAT
gi|301057067                  -AIIDITGGRKVMSAAAAMAAWQL-----KLELSYVEG-RF-DPHTRHP--IPGTDRLLI
gi|757183905                  -IYIDFTGGTKSMSAAAAMAGAMI-----DIQLVYVGTEDY-LVHFRKP--MPGSERLYY
gi|501216200                  -ILVDLTGGTKPMTVGLAKAAYVL-----GLGTVYIESDY----AQNRV--IPGTQRLIE
gi|501070122                  -IAVDLTGGKATMTVGLAKAAHVL-----RLASVYVDS----DYADNRP--IPGTQRLAT
gi|703489217                  -AYIDITGGRKVMSAAAALAAWQL-----DLRICYVES-DW-DPMLRRA--VPGSDRLLL
gi|559063203                  -VIIDITGGKKAMSAGAALAASQL-----DLPMCYIDS-TF-DPEMRQA--LPGSERLCV
gi|490153543                  -IYIDFTGGTKAMSAAAAMAGAMI-----DIQLVYVGTRNY-MNDFRKP--EPGSETLFY
gi|548231998                  -LHIDITGGTKAMSAAAAMAGAVI-----DIQLIYVGSNHY-LPDFRKP--CPGSETLFY
gi|656268823                  -IAVDVTGGLKPMAVGLEKAAHVL-----GLTTIYVESDYGPLPPDGRPGPLPGTQRLII
gi|497885042                  RVAIDITDGTKSMVTGCTLTANHL-----GMDLLYIHKEES-D--SGYL----GKEEPMI
gi|585249330                  -VILNVTGGKKVMSAAAALAAWQL-----DLELCYLDG-EV-EDAIGSV--IPGYQRLLL
                                    * *   *                     *                  *      

gi|499548365                  LPNPHEALAEVDALFAKELYGKGEFGQAAAYFRGMVGRTGNQA----YALYALLAEMYRA
gi|570755047                  LPNPHEALAEVDALFAKELYGKGEFGQAAAYFRGMVGRTGNQA----YALYALLAEMYRA
gi|499487817                  LPNPHEALAEVDALFAKELYGKGEFGQAAAYFRGMVGRTGNQA----YALYALLAEMYRA
gi|551068805                  LPNPHEALAEVDALFAKELYGRGEFGEAAGYFGRMVGKTGDQR----YLLYKMLAEMYHA
gi|516806813                  LPNPHEVLGGVDAFLARELYERGEFAKAQAYFGRLVESTGDRQ----YELYALLSGMYRA
gi|740203173                  LPNPHEVLAEVDALLARELYTKGEFGQAAKVFGEMGAKTGDRR----YELYAVLCQMYQR
gi|489138195                  LQDPHEVLGEVDALFAKELYGRGEFAQAARYFQKMVGATGDGR----WTLYQSLAEMYEA
gi|518381449                  LPNPHQVLADVDALFAREFYERGEYAKAADLFGAMVQKTLE-RR---FEVFGLLAGMYQA
gi|746416481                  LPNPHQVLADVDTLFAREFYERGEYAKAADLFGVMVQKTLE-RR---FEVFGLLAGMYQA
gi|503470028                  LPNPHEVVGEVDALFAKELYAKEDYGGAARYFSGLVGKTGD-QA---YTLYAMLAEMYAA
gi|740224592                  LPNPHEAVGEVDVLLAQELYEKGDFTGASGYFSGLVKQTGR-QE---YVLYATLSEMYGA
gi|746419752                  LPDPHEVGGEVDWLFASELYGKEDFAGAASYFNRLVGATGNQA----YTLYQNLAEMYRA
gi|740218035                  LPSPHEVLGDVDAFFALEHYSKGEFYEAQRYFRETAKKTKD-NR---YNVLADICVMYHA
gi|517272690                  LPNPHEVLGDVDEHLARSFYREGEFKKASAYFYETAKKTGQ-EG---FRLYGHLAKVYQS
gi|511098904                  LPNPHEVLGDVDEHLARSFYREGEFKKASAYFYETAKKTGQ-EG---FRLYGHLAKVYHS
gi|753938889                  LPNPHEVLGDLDEHLAQEFYKAREFGKAADRYNELRRKTGQ-GG---FEVYAALCEMYQR
gi|296849607                  LPNPHEVLGDLDEHLAQEFYKAREFGKAADRYNELRRKTGQ-GG---FEVYAALCEMYQR
gi|701167105                  LQSPHEVLADVDLLLALEHYEKGDYISAQRYFLQAREKTGS-KK---YEIYEDISLMYAF
gi|502941971                  INDPLGAFGDLEYQTAKALYNAQEYTLAAREFIRIADRTGDVRR---FSPYAKLSEAYAA
gi|648543053                  LENPREVIPDWLYNRGKEAYSQGDFARAKQNFSQAAEREGK--A---NSLEAILSEAYAH
gi|654417026                  LENPREVISDWIYHRAKDAYKRGDFSLAKQLFDQAKDHEGR--A---HSLEAVLAEAYES
```

```
gi|502777546                LENPREVIPDWIYHRAKDAYKRGDFSLAKQLFDQAKDREGR--A---HSLEAVLSEAYES
gi|654402559                LENPREIIPDWIYHRAKDAYARGDFSLAKQLFEQAKDREGR--A---HSLEAVLSEAYES
gi|302777911                VEDPYHVFGDLEKDKAIALFNKNEFVSAYNIFSDLEDRVADRD----YTFYKLLSNIYRL
gi|779961749                VEDPYHVFGDLEKDKAIALFNKNEFVSAYNIFSDLEERVADRD----YIFYKLLSNIYRL
gi|505124139                VEDPYHVFGDLEKDKAIALFNKNEFVSAYNIFSDLEERVADRD----YIFYKLLSNIYRL
gi|502759809                VEDPYEVFGDLEKDKAIALFNKGDFASAYKIFSELEERVAYRD----YTFYKMLSQIYSC
gi|655943915                VEDPYEVFGDLEKDKAIALFNKGDFASAYKIFSELEERVAYRD----YTFYKMLSQIYSC
gi|125715920                IDDPYTVFGDLEREQAISLFNKMDYVSAYRIFEELEQRVPGTK---EYSALKYISKAYNA
gi|489607383                IDDPYTVFGDLEREQAISLFNKMDYVSAYRIFEELEQRVPGTK---EYSALKYISKAYNA
gi|754095284                IDDPYTVFGDLEREQAISLFNKMDYVSAYRIFEELEQRVPGTK---EYSALKYISKAYNA
gi|489611620                IDDPYTVFGDLEREQAISLFNKMDYVSAYRIFEELEQRVPGTK---EYSALKYISKAYNA
gi|504021345                IDDPYSVFGDLEGEQAISLFNNMDYVSAYRIFKELEQRVPGTKA-KEYSALKYISKAYNA
gi|653611173                IDDPYSVFGDLEGEQAISLFNNMDYVSAYRIFKELEQRVPGTKA-KEYSALKYISKAYNA
gi|605572971                IENPYEVFGDIERDRAVTLFNGMDYASAAEIFDELRRKVPGLHE---FEALGLLSKAYDN
gi|740811186                IENPYEVFGDIERDRAVTLFNGMDYASAAEIFDELRRKVPGLHE---FEALGLLSKAYDN
gi|516314532                LDNPYDVFGDLEFTKAQALFRQLDYSSSKIILENLEHKTSTPDR---YKPLALLCSAYAH
gi|655011063                PSDPYEVFGDLEAAQARRLFNAHDYVNSERIYRDLAQRVKENKG---YEQLALLARAYAQ
gi|670513542                PSDPYEVFGDLEAAQARRLFNAHDYVNSERIYRDLAQRVKENKG---YEQLALLARAYAQ
gi|500595682                PEDPYTVFGDLEAAEARRLHNNHDYASAEKIFRDLAQRVPDNP---DYAIYADLSTAYLA
gi|301057067                LDDPTTLFGEQEMGRALEIFRAGAFEAARRRYDELCETLAVPVR---ARYMRALSEMYRA
gi|757183905                ISNPYEVFGDLEIEKALSLFGKYNYAGACEKLEILKDKVPDPAIRQQLEFVYQLAVTYEA
gi|501216200                PSDPYEVFGDLEAGEARRFFNAHDYVSAERIYADLARRVPAEDG-TFYACMAQLARAYTQ
gi|501070122                PEDPYLVFGDLEAAEACRLHNHHDYAGAERVFRDLARRVPDKQ---DYAIYADLSAAYLA
gi|703489217                LDNPTSLFGEQEMRAAQHSFDGGAFEAAHARFEDLAVRLAEPGR---ARFMAALSELYRA
gi|559063203                LPNPTALFGDKDLTAAMAMFGSGVYSGAHALFEKLSDSIAEPTQ---VRFLRDLAALYEA
gi|490153543                ISNPLAVFGDLEIEKAFALFEKYNYAGAQEKLAVLKESIPDPDTCRQLHLVYLLAKAYEA
gi|548231998                IANPLSVFGDLEIEKAFTLFRKKNYSGAREKLEYLKEVVPEPNIRQQLNFAYLLAKTYEE
gi|656268823                PEDPYVVFGDLEAAEAQRLYRKHDYAGAQRIFAALAERVPPPDG-RRYALLAQLAAAYAA
gi|497885042                LQDPLVVFGQNEYKRGIAKFNSEDFEAAAEIFKAIKERVINPRK---YEVLENLAVGYAH
gi|585249330                LDNPISIFGEQEMGRAAELFNAGAFDSASRRYLDLSERLAAPAA---ARFWKSASELYRA
                               *                                                   *

gi|499548365                WRALDFGEALKAGRKLL-GQLSQNVWLN---HPL-------NARREALEAQVALLEAVDR
gi|570755047                WRALDFGEALKAGRKLL-GQLSQNVWLN---HPL-------NARREALEAQVALLEAVDR
gi|499487817                WRALDFGEALKAGRKLL-GQLSQNVWLN---HPL-------NARREALEAQVALLEAVGR
gi|551068805                WRTLDAQGAVRQGRALL-GRLSENAWLQ---HPL-------NERRKALEAQVGLLEVVQS
gi|516806813                WQALDFSEALKQGKKLL-DRLSQDVWLN---HPL-------NRARGDLEAQVALLEGVER
gi|740203173                WYDLDFGGAAKEAKKLL-GRLSENIWLN---HPL-------NQNRGALEGQARLLEEVEG
gi|489138195                WHALNFPEAHKKGSGLL-DKLAQNAWLN---HPL-------NRSRDLLEKQVSLLEAGKA
gi|518381449                WYALDFAEAVRKGEALL-GRLEADALRQ---HPL-------NNRRGILEGQLSLLRAARD
gi|746416481                WYALDFAEAVRKGEALL-GRLEADALRQ---HPL-------NNRRGILEGQLSLLRAARD
gi|503470028                WQALDFKEAARKGQTLQ-RALEKDTWLQ---HPL-------GQHLSRLRRQVELLEAAAE
gi|740224592                WWALKFEEALRKGKSLL-ERLGNDPYLL---HPL-------NRQRPRLQAQVELLEEANR
gi|746419752                WRALDFKEAARKAESLL-EALRKNVWLQ---HPL-------NRHFQRLEGQASLLKGARD
gi|740218035                WYSLDIETAFEKSRKLV-EELQTDTWLN---HPL-------GKSRGFFETQSKILEAIKR
gi|517272690                WKDLDFPKAKKDLETLL-RDLDRDAHLN---HPL-------NARRKDLEAQQKGLSAILA
gi|511098904                WKDLDFPKAKKDLETLL-RDLDRDAHLN---HPL-------NARRKDLEAQQKGLSAILA
gi|753938889                WYALDFEGALSNAERLL-KFLSQDAYRN---HPL-------NLRAQRIKEQKEGLEGIVS
gi|296849607                WYALDFEGALSNAERLL-KFLSQDAYRN---HPL-------NLRAQRIKEQKEGLEGIVS
gi|701167105                WYNLNFQKAYKKSVQII-EKLNDEKHSK---HLK-------ILNYNYLFEQSELLKHMNE
gi|502941971                WLSNRFESAAHAFEGLF-SELDKPGFQT---HPL-------REQLPVLKQQHDGVLLLTK
gi|648543053                IDAAQFESAKTKLEELR-SLLQKPPYKQ---SPFAKYTNTIREQKEGLEEIIYLANSLSS
gi|654417026                LDAAQFKQAKDRLNDLL-ELLQKPAHRQ---SFLTKHTATIERQKEALEAVVQLTESLSV
gi|502777546                LDAAQFKQAKDRLNDLL-ELLQKPAHRQ---SFLTRNIATIERQKEALEVVVQLTEALSA
gi|654402559                LDAAKFGQAKDRLKELL-ELLQKPIHRQ---SFLTKYIAAIERQKEALEAVVQLTDALSS
gi|302777911                WDCLFFDECIREFEKLF-NTMKAWKYVE-KDLAA-------YKYYDTLFNQYKLIKPLES
gi|779961749                WDCLYFNECIKEFEKLF-SILKAWRYVE-KDLAA-------YKYYETLYNQYRLIKPLES
gi|505124139                WDCLYFNECIKEFEKLF-SIIKAWRYVE-KDLAA-------YKYYETLYNQYRLIKPLES
gi|502759809                WDNLSFNEAIEGFEKLF-KILKSWKPID-KDIIG-------YKYEEILYKQHELIKPLKE
gi|655943915                WDNLSFNEAIEGFEKLF-KILKSWKPID-KDIIG-------YKYEEILYKQHELIKPLKE
gi|125715920                WDSLDISGAADNLSKCY-EIVVTEGKID-KSFVL-------NSHIEKLEKQLGVIRVLEK
gi|489607383                WDSLDISGAADNLSKCY-EIVVTEGKID-KSFVL-------NSHIEKLEKQLGVIRVLEK
gi|754095284                WDSLDISGAADNLSKCY-EIVVTEGKID-KSFVL-------NSHIEKLEKQLGVIRVLEK
gi|489611620                WDSLDISGAADNLSKCY-EIVVTEGKID-KSFVL-------NSHIEKLEKQLGVIRVLEK
gi|504021345                WDSLDISKASENLSRCC-EIIETEGKLE-KGFTL-------SSHIEKLKKQLEVIKVLEK
gi|653611173                WDSLDISKASENLSRCC-EIIETEGKLE-KGFTL-------SSQIEKLKKQLEVIKVLEK
gi|605572971                WDSLNLAGAYDYMSKCC--EILSLLSLTGSATLL-------APHKSRLEQQLRLIGKLKD
gi|740811186                WDSLNLAGAYDYMSKCC--EILSLLSLTGSATLL-------APHKSRLEQQLRLIGKLKD
gi|516314532                WDDLKIKDSVTDLRLSK-DCLQRYLRLKTDSTIL-------SSYPTILEQQIQTLENLDL
gi|655011063                WDTFDLAAAAETMAQLLAQSLITELRVQ--QRLL-------EAQRDAINHLLSTMQAMNN
gi|670513542                WDTFGLAAAAETMAQLLAQSLITELRVQ--QRLL-------EAQRDAISRLLRTTQAMRY
gi|500595682                WDSFAPHQAGDALDRVL-ARADLPADLQPARSVL-------QAQRETLAQLTAINRRLTQ
gi|301057067                WCDLDLAALPAAVDALR--QALTQVRRD-----LG------VEHVDLLEEQVAFLSRL--
gi|757183905                WDALEFSKAYEAISKLY-KDMVRDRKIH-TDYIL-------MDFCELIEKQKEILEPMTK
gi|501216200                WESFDITTAVKTMADLLAHPLPARLALY--QSTL-------HDHYQALISLDATIKAMSN
gi|501070122                WDSFVPQPAAMALDRVL-AHNVLPDALQARRVTL-------EAQRQALTQLTAINQCLTN
gi|703489217                WCALELSALPTSIDRVH--ATLSPIRGS-----LS------VETTSSLERQLSFLSRL--
gi|559063203                WCNLDVDGLPELIARVR--EHLRENRMA-----LA------VPVTQRIAKQLEFAEAL--
gi|490153543                WDALDFLPAYEHMSALN-WQLRRDRKQH-RKFLM-------MDFCTLLEQQEVILSYLKE
```

```
gi|548231998      WDSLELSGAYQTILKLN-KELKRDKRIN-SRFLL-------MDKIDILKQQEEILAALSE
gi|656268823      WDVFDLPAAARDLQAVL-HEYAALPAAL--RATL-------VEQRAALAKLNQVGERLSK
gi|497885042      LESMRFDLAHDYIRKAV-------EWAE--RMELSEVPTY------DLRKQLKAIEPLMA
gi|585249330      WCDLDFEALSPLIENME-------RALADYRGPLP------ADVQRQISEQLKFLRCLDS


gi|499548365      F-LKARDFALKEG--------VYGLARTLLHLAQEAKEE-AAVLAALYAYRALELLLQER
gi|570755047      F-LKARDFALKEG--------VYGLARTLLHLAQEAKEE-AAVLAALYAYRALELLLQER
gi|499487817      F-LKARDFALKEG--------VYGLARTLLHLAQEAKEE-AAVLAALYAYRALELLLQER
gi|551068805      F-LASQDFGEKKG--------VYGLAWTLLRLSQAMADA-QPVLAALYAYRALELLLQER
gi|516806813      F-LRTRDFGELQG--------VLGLAATLLRLSERARGE-NPPLAALYAYRALELLLQER
gi|740203173      F-LRSGDFGQRRG--------ILGLTATLLHLAQREEAR-SLTLAGLYAYRALELLLQER
gi|489138195      F-LEGKDLGHRRG--------VGAVARTLLHLGE--KEH-RPLLAALYAYRALELLLQER
gi|518381449      F-LVGQDLG-NTLG-------VLGVVETLLRLSERSE-KTSLVLAALYAYRALELLLQER
gi|746416481      F-LVGQDLG-NTLG-------VLGVVETLLRLSERSE-KTSLVLAALYAYRALELLLQER
gi|503470028      F-LSTKDLG-KQRG-------VLGVAATLLHLSERTA-KKQPVLAALYAYRALELLLQER
gi|740224592      F-LDSKDLG-QRRG-------VLAVAATLLHLSEEEE---HPSLKALYAYRALELLLQER
gi|746419752      L-LTTGDLGNRRG--------VLGVAATLLHQSGRLADRDQTTLAALYAYRALELLLQER
gi|740218035      F-LEEKDYTRNKFG-------VFALAKTLLKKSEKHEMHGETILTALCAYRALELLLQER
gi|517272690      L-LEKKDLS-DKEG-------VAWLAATLLAGYE-GA-KGHLPLAALYAYRALELLLQHL
gi|511098904      L-LEKKDLS-DKEG-------VAWLAATLLAGYE-GA-KGHLPLAALYAYRALELLLQHL
gi|753938889      L-LKSQSFA-EKKD-------ILWLTATLLQLGDERK-ERQPVLAALYFYRALELILQHR
gi|296849607      L-LKSQSFA-EKKD-------ILWLTATLLQLGDERK-ERQPVLAALYFYRALELILQHR
gi|701167105      F-IETENYK-NKIG-------VIGLSETLLKLSKEASKNEELALSALYSYRALELLLQHR
gi|502941971      L-VDAYDDPAKRVATLSDAALVLWLLASLELMQSRFAEAGNFTAAGLLCYRSLEVTSQHR
gi|648543053      R---NIVPLAEPQK-------VAWALAALDFMAGRRQSAGRIAEAVLLRYRALEFFLQHR
gi|654417026      K-GEGIASLADPQK-------VACVLAALGFMSERRLKTGRLAEAVLLYYRALELFLQHR
gi|502777546      K-DKSIAPLADPQQ-------VACVLAALGFMTEKRLKTGRIAEAVLLRYRALELFLQHR
gi|654402559      K-GESIAPLEEPQK-------VACVLAALGFMAERRLKMGRIAEAVLLRYRALELFLQHR
gi|302777911      VNLNDKNEEWKYI---TNKDLYTPIIFSIYTNALRRSDEGKYDVAILLLYRILELMAQVR
gi|779961749      INLNDKNEEWEYI---TNKELYIPLMFSIYTNALRRSEEGKNDVAILLLYRVLELIAQVR
gi|505124139      INLNDKNEEWEYI---TNKELYIPLMFSIYTNALRRSEEGKNDVAILLLYRVLELIAQVR
gi|502759809      IDLKDKNKEWEYV---TNKHVYIPLLFSIYTNALRRSYEGKYDVAILLLYRILELIAQVR
gi|655943915      IDLKDKNKEWEYV---TNKHVYIPLLFSIYTNALRRSYEGKYDVAILLLYRILELIAQVR
gi|125715920      I-HCSEEAAKNKS---VIFDNIGYLIANLYQNAMRREKQEKYEMASLLLYRILEIVEQKR
gi|489607383      I-HCSEEAAKNKS---VIFDNIGYLIANLYQNAMRREKQEKYEMASLLLYRILEIVEQKR
gi|754095284      I-HCSEEAAKNKS---VIFDNIGYLIANLYQNAMRREKQEKYEMASLLLYRILEIVEQKR
gi|489611620      I-HCSEEAAKNKS---VIFDNIGYLIANLYQNAMRREKQEKYEMASLLLYRILEIVEQKR
gi|504021345      I-HCSEEATKDKS---IIFNNIGYLIANLYQNAMRREKQEKYEMASLLLYRILEIVEQKR
gi|653611173      I-HCSEEATKDKS---IIFNNIGYLIANLYQNAMRREKQEKYEMASLLLYRILEIVEQKR
gi|605572971      I-HEGQGHRQKDG---I-FHDISIVMANIYCNAMRKEKQHKYEMSSLLLYRLLEMVAQKR
gi|740811186      I-HEGQGHRQKDG---I-FHDISIVMANIYCNAMRKEKQHKYEMSSLLLYRLLEMVAQKR
gi|516314532      SLNELGENKTVAL---SKPNLYLPLMGTLRACAIRQEKRGKRDVATLLWYRLLELLSQQR
gi|655011063      S-HTALSTLANSD---A----ILPLLGSLHANALRREAQRRYDTAALLHYRSLELMSQHR
gi|670513542      S-NTDLSTLADAD---A----ILPLLGSLHANALRREAQRRYDTAALLHYRSLELMSQHR
gi|500595682      R-KTPPADALTAL---RDLNQGLALLGSLHSAALRRAAQERYDVAALMRYRCLELLSQHR
gi|301057067      T-------TGDRNA----------TLLCFYVLGVHYQQLARHDFAALLFYRTIEGCLTRR
gi|757183905      I-LGFIKDKKNMKV-LQDQTYIVPLMFTMYNNAWVREEQEKYDSSTLLLYRLLEMISQRR
gi|501216200      Q-QQALTTLANRQ---A----ILPLLGSLYANALRRETQRRYDTAALLRYRCLELMSQHR
gi|501070122      R-HSQAADALAAL---NDLDQVLALLGSLHGAALRRAAQERYDVAALMRYRCLELLSQHR
gi|703489217      A-------RRDRSA----------LLLGFHILDDHYRGIGRHDFAALLSYRTIEGCLVER
gi|559063203      A-------GRDGPT----------MLLNFFLLGEHYRAQGRHDFAALLYYRSIEKVFEER
gi|490153543      I-PLLMKDKKNKEI-LKNKDIITALMFTMCQNGLVREQQEKYDMATLLFYRLLEMIEQRR
gi|548231998      I-PQYIAEKRNAEL-LQKEKYIVPLMFTMCQNAMIREEQEKLDMATLLLYRLLEMIEQKR
gi|656268823      H-DTRLAVLQDKQ---A----VLALLGTLHANALRRQEQARYDVAALLRYRCLELIGQQR
gi|497885042      L-----HDRSDREK-LSNDQLYWHLYAYLFEMTKHYKSNNKHDITALLTYRCLEMTVQRL
gi|585249330      T-------NASRLD----------LMLAFHLLGDHYTAVGRHDFAALLYYRTIEGCLAWR
                                                                *  **  *

gi|499548365      LA-LL-GRRAEAPGLS---PEEA---EALRKAL------AELLGV-LPE-E---V-RLP-
gi|570755047      LA-LL-GRRAEAPGLS---PEEA---EALRKAL------AELLGV-LPE-E---V-RLP-
gi|499487817      LA-LL-GRRAEAPGLS---PEEA---EALRKAL------AELLGV-SPE-E---V-RLP-
gi|551068805      LA-LL-GRRAEAPGLS---LEEE---AATREEL------ARILRL-SPG-E---A-RVG-
gi|516806813      LS-RL-GRRAEAPGLT---PEEA---QGLREVL------AGFLQI-PPE-A---V-EVP-
gi|740203173      LS-RL-GRRAEAPGLS---QEEQ---EGLRREL------AALLSL-PEG-E---V-RVS-
gi|489138195      LY-RY-GRLADQPNLT---PEEE---AALRNAL------AEILGE-PPS-A---V-EVP-
gi|518381449      LF-LH-GRRADSPALT--PPEEG----ALRQEL------ARLLRVHEEAVRL--------
gi|746416481      LF-LH-GRRADSPALT--PPEEG----ALRQEL------ARLLRVHEEAVRL--------
gi|503470028      LYRFD-RRRAETPNLS--PEEEA----ALRDEL------AALLPDTDE-IRV--------
gi|740224592      LH-RH-GRRAEDPRLS--PEEKE----ALGQEL------ARILGGE---ARV--------
gi|746419752      LR-RY-GRLAEAPELS---PEER---EALQGEL------GRIL---PGE-E---VPRVR-
gi|740218035      LS-LY-NLTPDMP-LT--DEDKI----AIRREM------SKILQKPEDQVEM--------
gi|517272690      AA-GM-GLDLEAPRPT--PEEEE----ALKATL------KALLPTEGE-IRI--------
gi|511098904      AA-GM-GLDLEAPRPT--PEEEE----ALKATL------KALLPTEGE-IRI--------
gi|753938889      IA-VN-GRSNDNPVLT--PEEQD----HLRQSL------ARWLKRSLEEIKP--------
gi|296849607      IA-VN-GRSNDNPVLT--PEEQD----HLRQSL------ARWLKRSLEEIKP--------
gi|701167105      IH-EL-DKPIETEKIP--SQELE----MLKQKL------STLLNQPPDSIKF--------
gi|502941971      LA-TY-RFDSLAANYPALPVPQD----VLQDAY------TAAWQKVASKKQAASPRPLPP
gi|648543053      LA-QR-GFDTAKPDFQKLCETVQ----MSLEDL------KIKYQVERNGAGLK-TEDKLE
```

```
gi|654417026                    LA-LR-NFDTAKPDFDRLCAEAG----ITIQEL------NDRYQEECRAARAR-LGGALQ
gi|502777546                    LA-LR-GFDTEKPDFPGLCAANH----TSIEDL------QEKYQAERKAAKAG-SDDKLI
gi|654402559                    LA-LR-GFDTANPNFRGLCAAAH----ITIEDL------IEKYQGERRAARAK-PDDELE
gi|302777911                    LA-KY-GFNVSSPDYSFFSVGEQ---ELLSRMN------ENIKSF----KNFKTYTELP-
gi|779961749                    LA-KN-GFNVSLPDYSVFNIDKH---ELLSRMN------ENIKHF----KNFKTYAELP-
gi|505124139                    LA-KY-RFNVSSPDYSVFNIDKH---ELLSRMN------ENIKHF----KNFKTYAELP-
gi|502759809                    LA-SY-GFSVSLPDYSKLPVGGN---ELLEKMN------ENIKPFRKRSKNFKNYSELP-
gi|655943915                    LA-SY-GFSVSLPDYSKLPVGGN---ELLEKMN------ENIKPFRKRSKNFKNYSELP-
gi|125715920                    LW-NY-GVDTSDADFTKLCEDEK---VLLEKAN------KIIRSV-KGFNE---WKQLD-
gi|489607383                    LW-NY-GVDTSDADFTKLCEDEK---VLLEKAN------KIIRSV-KGFNE---WKQLD-
gi|754095284                    LW-NY-GVDTSDADFTKLCEDEK---VLLEKAN------KIIRSV-KGFNE---WKQLD-
gi|489611620                    LW-NY-GVDTSDADFTKLCEDEK---VLLEKAN------KIIRSV-KGFNE---WKQLD-
gi|504021345                    LW-NY-GIDTSNADFTKLCEDES---ILLENAN------KIIRKI-KGLNE---WKQLD-
gi|653611173                    LW-NY-GIDTSNADFTKLCEDES---ILLENAN------KIIRKI-KGLNE---WKQLD-
gi|605572971                    LW-NL-KIDTEDAEYAETGFEPQ---ALLDRVN------ALRTRV-KGFHP---IEKLD-
gi|740811186                    LW-NL-KIDTEDAEYAETGFEPQ---ALLDRVN------ALRTRV-KGFHP---IEKLD-
gi|516314532                    LS-TY-GLDTKCPDYNNSQLQNKISSDSLLEKYITCGLSTSKKGV-KPENM----TELP-
gi|655011063                    LA-TH-GVLAERPDLDRL-KQRQ---PQLENEY------RAVERS-LGFRERGLQPNRDG
gi|670513542                    LA-TH-GVLAEHPDLDRL-KQRQ---PQLENEY------RAVERS-LGFRERGLQPNRDG
gi|500595682                    LA-TY-GIWTAEPSFDAALRRVP---D-LDDRY------RQAQRD-QGFRK---QYPLPS
gi|301057067                    LQQRFPGLGDDRIDWALFGDPAA----ARQRYS------RVAGELGAGST------TPP-
gi|757183905                    LS-LY-NLNVSKAEYLSMKYPNK---PEYDKKEAVEKLEVFKRKVLEIKKQIF-TRCDDY
gi|501216200                    LA-TR-GVWVEKPDLDQL-KAQI---PDLDRSY------RAVERT-LGFRERGLQPNRDG
gi|501070122                    LA-TY-SVWTAAPSFDAALQRVP---D-LNQQY------RQAQCE-QGFRK---VYDLP-
gi|703489217                    LKVRFPGFDDSDPEYDLISTDQQ----QFKRSW------TDVMNLVDGQQKRR---SLP-
gi|559063203                    LSSEF-GLDPVDPDYTKLGDVED----LTTRYA------ALTTEVYGAPTD-----TLP-
gi|490153543                    LA-EY-NLFVSGMNYKKVKYNLKRA-PEFAQLSSKDRYDLLKQKVCEIKKQLFGTGESPR
gi|548231998                    LA-GY-NLYVSKMNYKEIKVNIQIH-PEWDGIDAKELFRQIKERYLDMKIQLFGKTGNTY
gi|656268823                    LA-EY-GILSERPNFDRF-GAGR---SALEQRY------RQVQKS-VGRKQCYPLPER--
gi|497885042                    LL-KH-GIYTANASFSHLDQQQL-----LKDYNVL------------GEQVFPNYRPLHY
gi|585249330                    LENKYAGFRCDEPDYSLLGEVGE--------EY-------ARVSAGMPGKESG----LP-


gi|499548365                    --AKLGLLDLLAFLRLKGDEALGRLS----LAELRGLAGALKGRNSALLVHGFDVPSPKA
gi|570755047                    --AKLGLLDLLAFLRLKGDEALGRLS----LAELRGLAGALKGRNSALLVHGFDVPSPKA
gi|499487817                    --AKLGLLDLLAFLRLKGDEALGRLS----LAELRGLAGALKGRNSALLVHGFDVPSPKA
gi|551068805                    --AKLGLLEILAFLRAHGDSVVGGLP----LAELQGLSGVLKARNEALLVHGFRVPTQNE
gi|516806813                    --PKLGLLHLVAFLRLKGDSLLATLP----QERLMGLSGVLKARNESLLVHGFLVPREKE
gi|740203173                    --PKLGLLDLVAFLRLQADPLLAREP----LERLRGLSGALQARNASLLVHGLGRPSDKD
gi|489138195                    --KKLGLLHLIGLLRLLGDPLLAGKP----AGELRGLGGVLQARNTSLLIHGFEVPTGRQ
gi|518381449                    -GAKLGLLDLVAFLRILGDPVLASQ----KVEALQGLAGVLKARNDALLIHGFAVPSEKE
gi|746416481                    -GAKLGLLDLVAFLRILGDPVLASQ----KVEALQGLAGVLKARNDALLIHGFAVPSEKE
gi|503470028                    -GPKLGLLEVLAFLRVLGDPVLKKK----GVKELQGLAGVLKARNQALLIHGLEVPSEKQ
gi|740224592                    -GDRLGLLDVMAFLRVLGDPVLKGM----SLQEVQGLKGVLQARNQSLLVHGLEVPTEKE
gi|746419752                    --EKLGLLEVLAFLRVLGDPLLASWN----VRDLQGLAGVLKARNQALLVHGLQVPSARQ
gi|740218035                    -HDKLGLFGMAILLIVKKDSCVQGIF---DQKRLKALSLALQSRNSSLLIHGFDFPNEKQ
gi|517272690                    -PERFGLLHLLAYLKAKDHPAFRHL----DSKRLQGLQGALRARNKALLIHGLEGPKEGD
gi|511098904                    -PERFGLLHLLAYLKAKDHPAFRHL----DPKRLQGLQGALRARNKALLIHGLEGPKEGD
gi|753938889                    -ITKLGLLESMALLRYLEDPLLDNF----SENDLQGYQGMLQSRNKSLLIHGLEVSKKGD
gi|296849607                    -ITKLGLLESMALLRYLEDPLLDNF----SENDLQGYQGMLQSRNKSLLIHGLEVSKKGD
gi|701167105                    -KPKLGLLELVVYLSILNDDAVKEVF---DQESIKKLCWNLEARNASLLIHGLKTPSERE
gi|502941971                    QGQPVPLFESYLLLGALEDPLVKAL-----VHRQNDLMGLAELRNQSLWIHGYKPISEKA
gi|648543053                    QKTDVDFVTAFFLLRALGDNAAQAV-------NPNKLVGLAKARNSSVFAHGFEPPKEGM
gi|654417026                    QKIAVDLITAFFLLRALGDEPALAV-------NANKVLGLSSARDNSIFAHGFLLPTKAN
gi|502777546                    EKNAVDFITAFFLLRALGDGPALAV-------NVNKVLGLTKTRNASVFAHGFTLPTEDN
gi|654402559                    QKSAIDFTTAFFLLRALDDEPALAV-------SANKVLGLASARDISVFAHGFVLPTEAN
gi|302777911                    -QNSMALFDAYVLIKAIEDELMEGIN-------IGLIYSNVQLRNKSIFTHGFGMLSNKD
gi|779961749                    -NNSIALFDAYVILKAIEDELMEGIN-------IGMIYSNVKLRNKSIFAHGFGILSNND
gi|505124139                    -NNSIALFDAYVILKAIEDELIEGIN-------IGMIYSNVKLRNKSIFAHGFGILSNND
gi|502759809                    -QSSISLFDAYVLLKAIEDKLVKDIE-------LGLIYRKVKLRNKSIFAHGFGILMEKD
gi|655943915                    -QSSISLFDAYVLLKAIEDKLVEDIE-------LGLIYRKVKLRNKSIFAHGFGILMEKD
gi|125715920                    --KKISLLAGYILLAAVGDDIIKTKKPGKEIDSINRLRNKVEARNNSIFAHGYEFISKEK
gi|489607383                    --KKISLLAGYILLAAVGDDIIKTKKPGKEIDSINRLRNKVEARNNSIFAHGYEFISKEK
gi|754095284                    --KKISLLAGYILLAAVGDDIIKTKKPGKEIDSINRLRNKVEARNNSIFAHGYEFISKEK
gi|489611620                    --KKISLLAGYILLAAVGDDIIKTKKPGKEIDSINRLRNKVEARNNSIFAHGYEFISKEK
gi|504021345                    --RKISLLAGYIILAAVGDEIMKTKKPGKETDSINRLRSKVDVRNNSIFAHGYEFISKEK
gi|653611173                    --RKISLLAGYIILAAVGDEIMKTKKPGKETDSINRLRSKVDVRNNSIFAHGYEFINKEK
gi|605572971                    --KQISLIAGFILLAALEDDIIRTKKPDAVIGRIESLKNKVDLRNKSIFAHGFTFIDADK
gi|740811186                    --KQISLIAGFILLAALEDDIIRTKKPDAVIGRIESLKNKVDLRNKSIFAHGFTFIDADK
gi|516314532                    --NIIDLINGYKLLKALDDPLAIDQD-------LNRITNQVNIRNNSIFAHGFKPLNDNS
gi|655011063                    QYNPITLLNGYMLLKALRDPLVRSIN-------LADIQNRVSVRNKSILAHGFRQITETE
gi|670513542                    RYNPITLLDGYMLIKALQDPLVQSVN-------LADIQNRVSVRNKSILAHGFRQITETE
gi|500595682                    SERSIALFDGYMLLQALDDPLVRGW-------NIGDIRQRSYVRNTSILAHGFRPISSLE
gi|301057067                    --SRLMLMSSVKLLCAYDDELTRRCG-LDDADGIRGLEDQTKKRNKSVLAHGFGTVTVGE
gi|757183905                    LQPQVALLEGYIYLVALEDTLMGTNT-KSRLNYLKKLRSMVFLRNNSIFAHGLAPVSKED
gi|501216200                    QYSSITLLNGYMLLTALSDPLIQTIT-------LTDIRQRVNVRNKSILAHGFRQITEAE
gi|501070122                    -ERSIALFEGYMLLQALDDPLVREW-------DIGLVRQRSYVRNTSILAHGFRAISRHE
gi|703489217                    --PFIGLFSAAVVLRVLDDDLLRLAG-LWEVDELRSLRDLARVRNDSVLAHGDRSVSKTD
gi|559063203                    --RKIALMDAMLLLCLKDDAVLKRIG-WTTPSSISSMRGVVDTRNRSVLAHGTASVSMDQ
```

```
gi|490153543                  LQKQVSLMEGFVILHALGDPITADPA-GESVERLEQIRKMVFLRNHSIFAHGLGPVGFQD
gi|548231998                  MPEQISLLEGFILLAALDDEMLVRGN-SSVIDKLKRIRAMVYLRNNSIFAHGLGPVSQTD
gi|656268823                  ---AFGLFVGYMLLKALEDELVHDLS-------IGMIEARSAARNTSMLTHGFRLIDQVE
gi|497885042                  FPNKITLMSGLILLRALQEPMVRDIG-------LADMNNMITLRNKSRLVHGFELLTEEQ
gi|585249330                  --AKVSLVEAAKLLVALKDGFAVATQ-LAKGSGIVSLASLAATRNHSILAHGFKQIAPSD
                                                                         *      **

gi|499548365                  VEGIARLAQGLLQDLE--ARTALGPL-S-PE----PVP-------LGF--------
gi|570755047                  VEGIARLAQGLLQDLE--ARTALGPL-S-PE----PVP-------LGF--------
gi|499487817                  VEGIARLAQGLLQDLE--ARTALGPL-S-PE----PVP-------LGF--------
gi|551068805                  VGQLQRLAQRLLEDLE--ARSGVRGV-P-WD----PVP-------LGF--------
gi|516806813                  LQSLLSLAQRLGEDLA--A--GARP--S-LE----PVP-------LGF--------
gi|740203173                  LEQVRKLARSLLEGLQ--REVGPAP--R-LA----PVE-------LSF--------
gi|489138195                  VGALKGLAKDLLADLD--KELGPAV--S-LE----PLP-------LGF--------
gi|518381449                  LKQIRNLLQPLLADLR--ERAGLRVS-------LHPVA-------LGSTF------
gi|746416481                  LRQIRNLLQPLLADLR--ERAGLRVS-------LHPVA-------LGSTF------
gi|503470028                  VKQVQTALRVLLGALQ--EELAYRTD-------LKTIP-------LERV-------
gi|740224592                  VGKVAKGAKGLLRALQ--DELRLQPS-------LEPAP-------LEFWGRREE--
gi|746419752                  VEQVAGLARKLLQDLQ--REVGLQP--D-LEAI--PLP-------R----------
gi|740218035                  VEYIKECARKLLKDLE--IRAELRLD-PSIDMCFEKAG-------LDLFKW-----
gi|517272690                  VDQVARLARELLKGLG--VEAK-----------AEPIP-------L----------
gi|511098904                  VDQVARLARELLKGLG--VEAK-----------AEPIP-------L----------
gi|753938889                  VEKLQQFARKLYLTTR--EEAKLALS-------VEPVE-------LM---------
gi|296849607                  VEKLQQFARKLYLTTR--EEAKLALS-------VEPVE-------LM---------
gi|701167105                  NERLQEKAEKLAKTIK--KSEDVSFS-------VEALK-------IDKGILRFEEL
gi|502941971                  YRKLASLLSPLHEALL--QLEGRSER---CPASAVPLP------------------
gi|648543053                  ANDLAQVLQTLV------ERGGLPKV-E-----YSPIP-------LA---------
gi|654417026                  ADNLSEVLTDLV------RKGGLSEV-R-----FEPIP-------LP---------
gi|502777546                  AKNLSEVLVALV------QSGGLPEV-R-----FEPIP-------LS---------
gi|654402559                  AKNLSEVLMALV------QGGDLPEV-K-----FEPIP-------LA---------
gi|302777911                  FDKIRKTVKRIFTSFY--EIEGIDF-------------------------------
gi|779961749                  FDKFHEIVRKIFIRFC--DLENINFE-S-ICGNYKFIL-------LS---------
gi|505124139                  FDKFHEIVRKIFIRFC--DLERINFE-S-VCSNYKFIL-------LS---------
gi|502759809                  FNDFHETVKKILIRFC--EIERIEFE-K-VCKDYKFVL-------LE---------
gi|655943915                  FNDFHETVKKILIRFC--EIERIEFE-K-VCKDYKFVL-------LE---------
gi|125715920                  YNEFKKVVEDYMNLLC--SIEGIDKE-E-LFDSCEFIK-------L----------
gi|489607383                  YNEFKKVVEDYMNLLC--SIEGIDKE-E-LFDSCEFIK-------L----------
gi|754095284                  YNEFKKVVEDYMNLLC--SIEGIDKE-E-LFDSCEFIK-------L----------
gi|489611620                  YNEFKKVVEDYMNLLC--SIEGIDKE-E-LFDSCEFIK-------L----------
gi|504021345                  YMEFKKVVEDYIDLLC--SIDCINKE-E-IFSICKFIN-------L----------
gi|653611173                  YMEFKKVVEDYIDLLC--SIECINKD-E-IFSICEFIN-------L----------
gi|605572971                  YSQFKAAVDEYVDRFF--HLEELDRD-E-AFGPMNFIT-------LSAD-------
gi|740811186                  YSQFKAAVDEYVDRFF--HLEELDRD-E-AF-------------------------
gi|516314532                  FMSFKTLVESLVDIFK--TIQPENQA-Y-WNDCI-FVE-------EIV--------
gi|655011063                  YRDFVSVVEQVLDQFF--ITTKHRRE-D-WEEQYQFVK-------L----------
gi|670513542                  YRDF----------------------------------------------------
gi|500595682                  YEQFADIVEELLDRFF--ALIGRSRQ-E-WERVHRFVS-------LAA--------
gi|301057067                  TEELRRRADSLLQVYW--ELHGDTGSLDEMCDRLQFVRTDR---------------
gi|757183905                  FTRFKNFVILLFQRFC--AIEEIDFA-Q-ISEDILWINPLESKYCMHVDLLHI---
gi|501216200                  YREFARVVEQLLDQFF--TIAQEQRS-S-WEERYRFIE-------LPAT-------
gi|501070122                  YEQFADIVENVLDRFF--TIAQLPRY-E-WEQTYHFVT-------LAV--------
gi|703489217                  SEQLNSMAQSVVRAYW--TLHHADENLDELRARLRFIRAGR---------------
gi|559063203                  SAQLSGRAGALLRYFL--QVHAPNQDITERIKTLRFVSEL----------------
gi|490153543                  FHKFQCFVLDLFQLFC--SLEQIPYQ-E-YRENMTWVNPLTSKYDTGM-GE-----
gi|548231998                  YLKFKSFVLELFREFC--DIEQISFA-Q-YNSKMTWITPFDSVYYSGLEGN-----
gi|656268823                  YESFCEVVEQVLERYL--QLVCVSRA-D-WETTFRFVA-------IDVLTNPEE--
gi|497885042                  VEGFYNRIKGLSNFVWNHDKHNLKVEVG-FEEFSSPYSFVN--ISLRSRLVSV---
gi|585249330                  SQKLGERAAFFLKKLW--ELERPSDNLDELRLNLAFVRQGT---------------
```
