# Supplementary material for: Structural basis for the endoribonuclease activity of the type III-A CRISPR-associated protein Csm6
Source: RNA. 2016 Mar;22(3):318–29. doi: 10.1261/rna.054098.115 (PMC4748810; doi:10.1261/rna.054098.115)
Supplement: Supplemental Material [file supp_054098.115_SuppMaterial.pdf]

Revised Supplemental Material for:

Manuscript #RNA/2015/054098

Structural basis for the endoribonuclease activity of the type III-A CRISPR-associated protein Csm6

Ole Niewoehner[1] and Martin Jinek[1*]

[1]Department of Biochemistry, University of Zurich, Zurich, Switzerland

Running title: Crystal structure of Csm6

Keywords: CRISPR, Cas protein, ribonuclease, Csm6, crRNA, HEPN domain

[*]Correspondence to: Martin Jinek, Department of Biochemistry, University of Zurich, Winterthurerstrasse 190, CH-8057 Zurich, Switzerland. E-mail: jinek@bioc.uzh.ch

SUPPLEMENTAL FIGURES

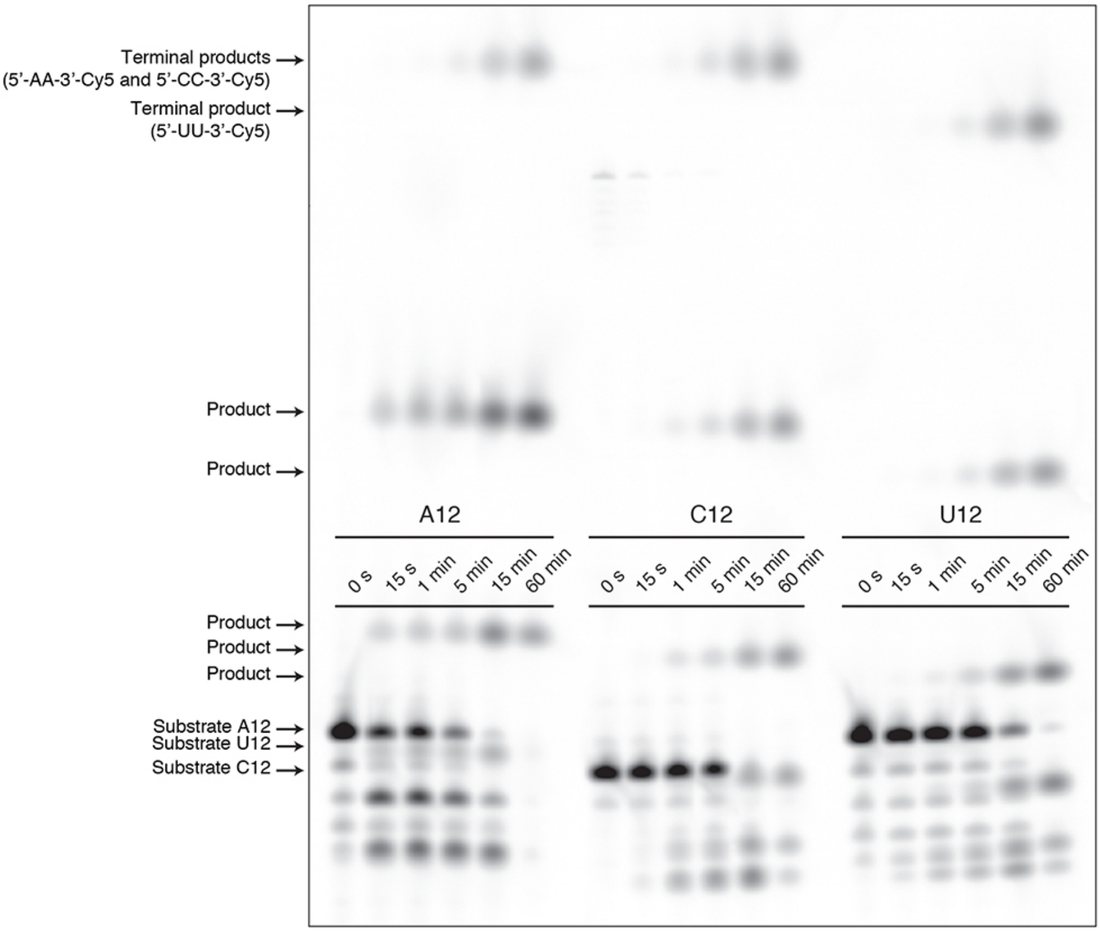

Supplemental Figure 1. The ribonuclease activity of TtCsm6 is base-unspecific

Synthetic 12-mer homo-oligonucleotides (A_{12}, C_{12} and U_{12}) labeled with the Cy5 fluorophore at their 3' ends were incubated with TtCsm6 at 37 °C and the reactions were sampled at indicated time points. Cleavage products were resolved by electrophoresis on a 16 % denaturing polyacrylamide gel and visualized using a fluorescence gel scanner. Due to the size and charge of the Cy5 fluorophore, short oligonucleotide products have anomalously low electrophoretic mobility (Killelea et al. 2014).

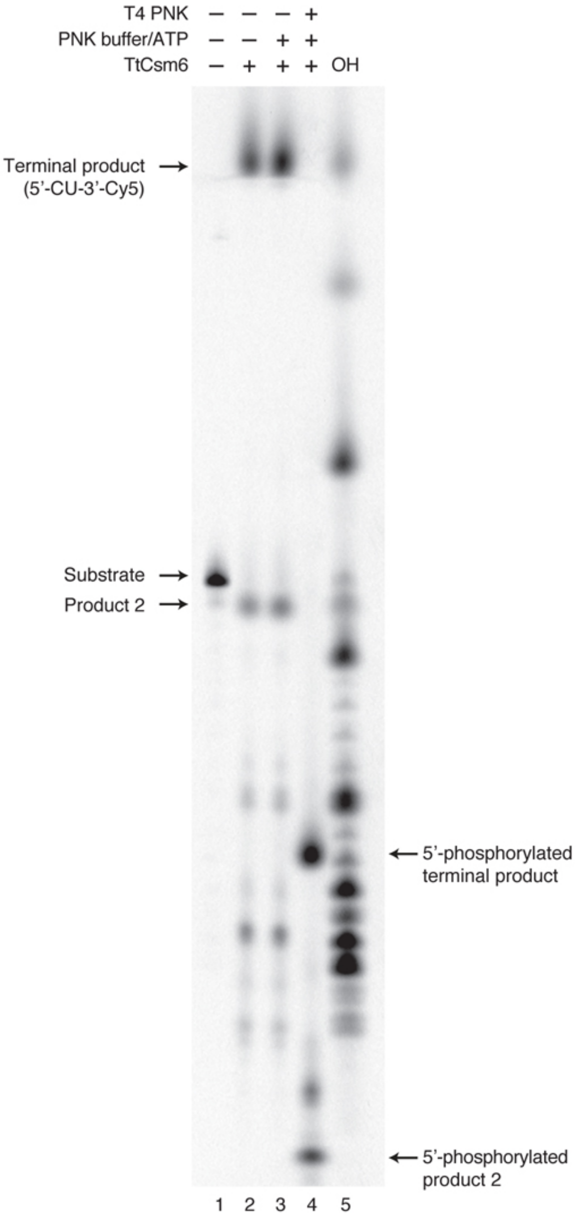

Supplemental Figure 2. TtCsm6-catalyzed RNA hydrolysis yields products containing a 5'-hydroxyl group.

A 24-mer RNA oligonucleotide (ACUGCAACGCAAUAUACCAUAGCU) labeled with Cy5 at its 3' end (lane 1) was digested with TtCsm6 for 15 min at 37 ^{o}C (lane 2), yielding a terminal product band that most likely corresponds to 5'-CU-3'-Cy5 (Killelea et al. 2014). The reaction mixture was subsequently incubated in the presence of ATP (lane 3) or ATP and T4 polynucleotide kinase (T4 PNK, lane 4). Alkaline digest of the RNA substrate served as a control (lane 5). Treatment with T4 PNK results in marked shifts in electrophoretic mobility of the cleavage products, indicating that the products of TtCsm6-catalysed RNA hydrolysis carry a free 5'-hydroxyl group.

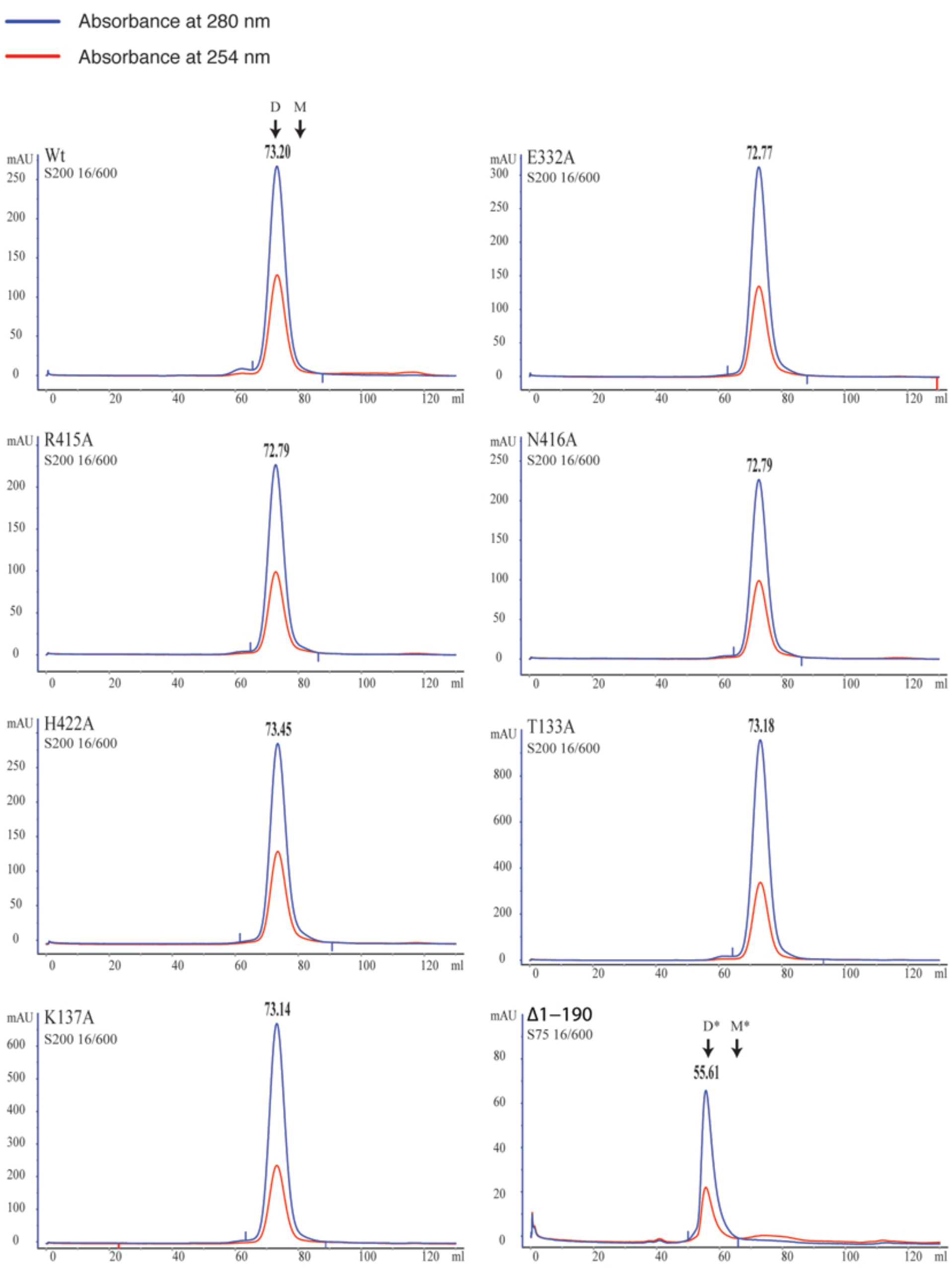

Supplemental Figure 3. Wild-type and mutant TtCsm6 proteins exist as homodimers in solution.

Size exclusion chromatograms of wild-type and mutant full-length TtCsm6 proteins and a truncated TtCsm6 lacking the N-terminal CARF domain (Δ1-190). Red and blue lines indicate absorbance at 254 nm and 280 nm, respectively. Black arrows indicate expected elution volumes for dimeric (D, 72.8 ml) and monomeric TtCsm6 (M, 80.6 ml), as well as Δ1-190 dimers (D*, 56.5 ml) and monomers (M*, 65.6 ml). Full-length TtCsm6 proteins were resolved on a Superdex 200 16/600 column (GE Healthcare) while the Δ1-190 mutant was resolved on a Superdex 75 16/600 column (GE Healthcare). Elution volumes were calculated based on a standard curve generated using a set of gel filtration standards (Bio-Rad).

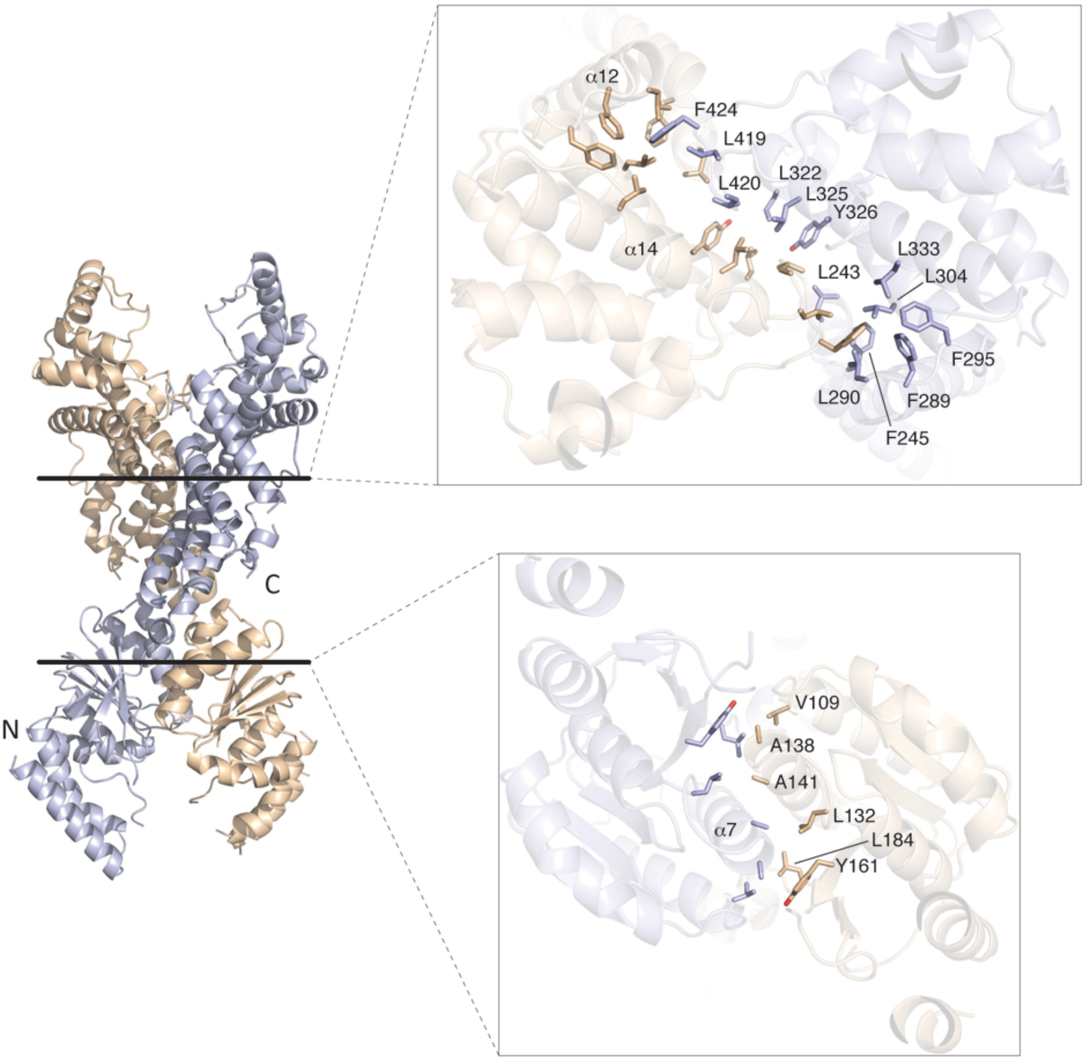

Supplemental Figure 4. Dimerization interfaces in TtCsm6.

Left: Cartoon diagram of the TtCsm6 dimer. Thick lines indicate sections through the dimer depicted in insets. Top inset: dimer interface of the 6H and HEPN domains viewed down the dyad axis of the dimer. Hydrophobic amino acid side chains involved in dimerization are shown in stick format. Bottom inset: dimer interface of the CARF domains viewed down the dyad axis.

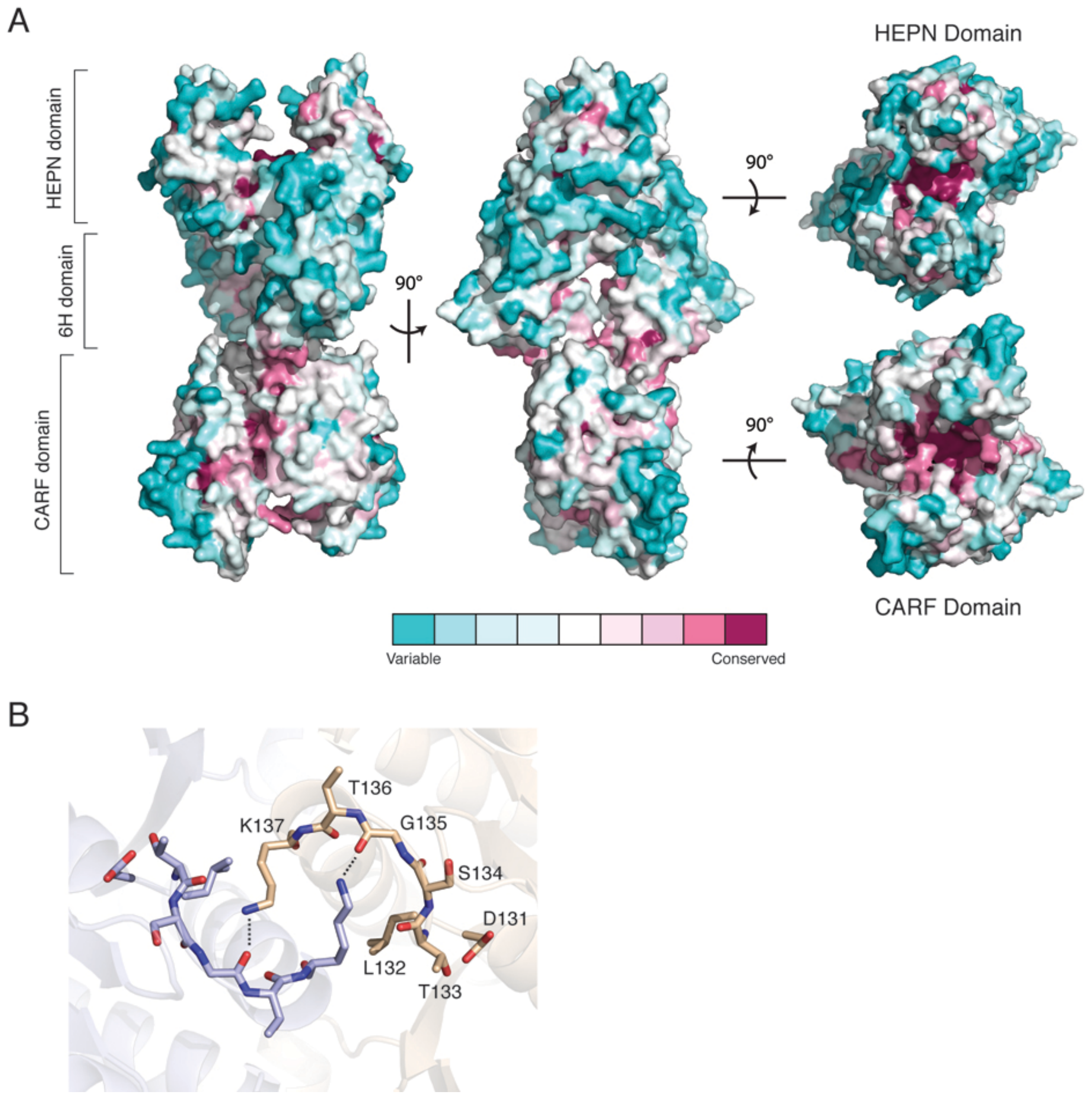

Supplemental Figure 5. The TtCsm6 dimer features two conserved surface pockets.

(A) Surface representation of TtCsm6 colored according to evolutionary conservation. The color scheme was generated using the Consurf server (Landau et al. 2005) based on the multiple sequence alignment of TtCsm6 with 50 closest homologs (Supplemental Data File 1). The dimer is shown in the same orientations as in Fig. 2A. *(B)* Zoom-in view of the conserved pocket at the CARF domain dimer interface. The conserved (D/N)-X-(S/T)-X_3-(R/K) motif (Asp131–Lys137) is depicted in stick format.

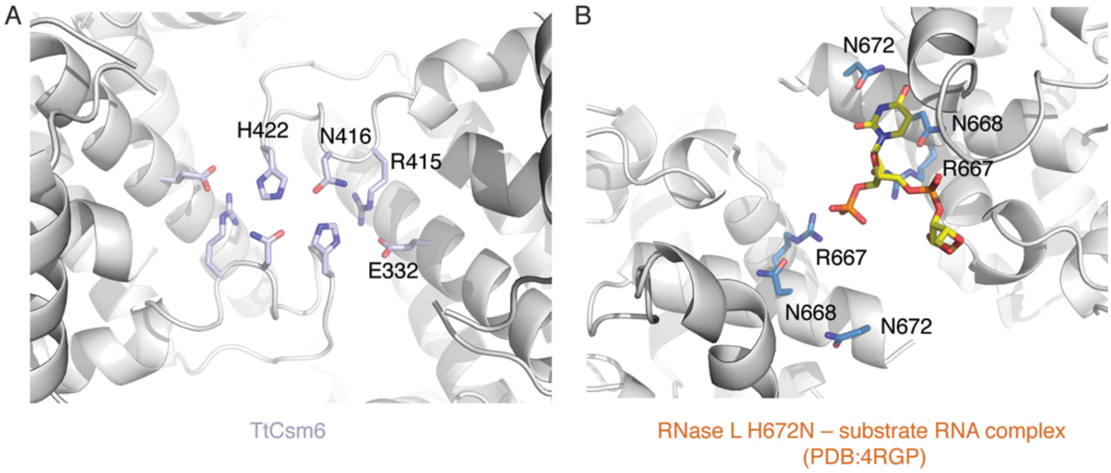

Supplemental Figure 6. The composite ribonuclease active center of TtCsm6 resembles the active site of RNase L.

(A) The active site of TtCsm6, viewed down the dyad axis of the TtCsm6 dimer. Invariant active site residues are shown in stick format. The Ni^{2+} ion present in the TtCsm6 structure has been omitted for clarity. *(B)* Active center of RNase L as observed in the structure of the H672N mutant of RNase L bound to a substrate RNA (PDB ID 4RGP). Invariant active site residues and the RNA substrate are shown in stick format.

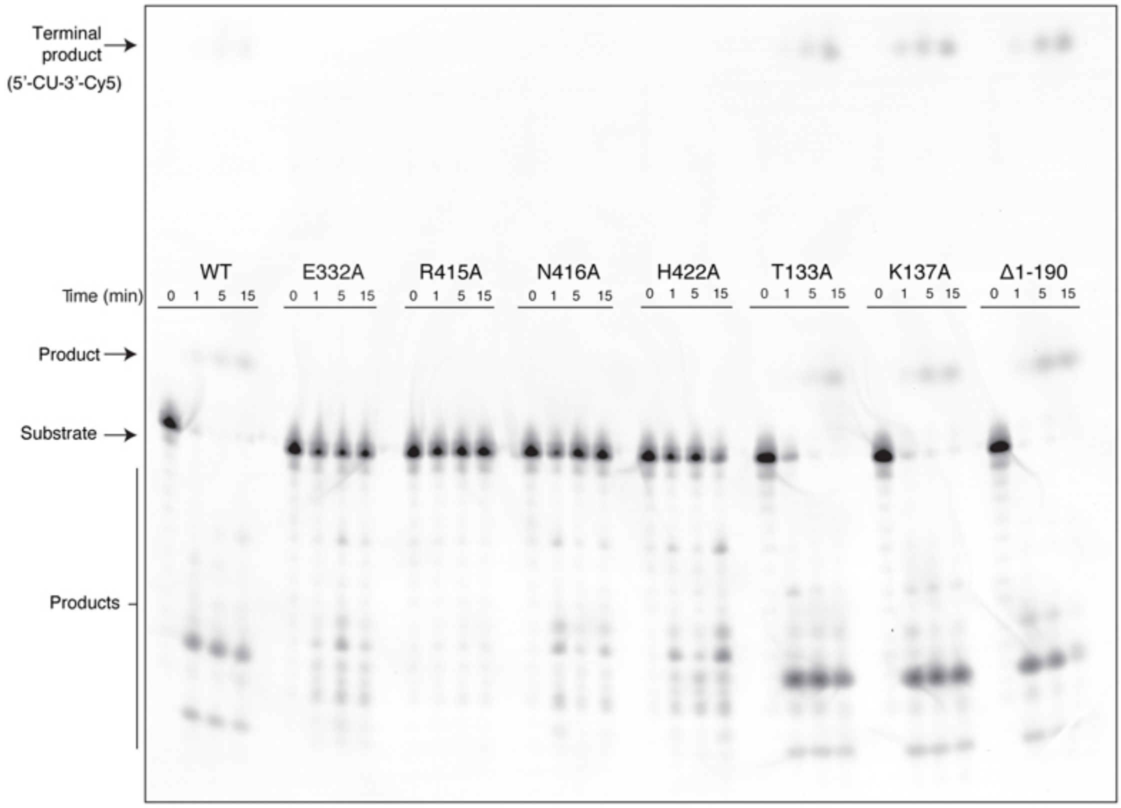

Supplemental Figure 7. The C-terminal HEPN domain of TtCsm6 harbors the endoribonuclease active site.

Nuclease activity assay of TtCsm6 proteins. The image represents a full-size scan of the image shown in Fig. 3C. The assays were performed using a 24-nt ssRNA substrate labeled with Cy5 at the 3'-end. Reactions were resolved on a 16 % denaturing polyacrylamide gel and visualized using a fluorescence gel scanner. Due to the size and charge of the Cy5 fluorophore, short oligonucleotide products have anomalously low electrophoretic mobility (Killelea et al. 2014).

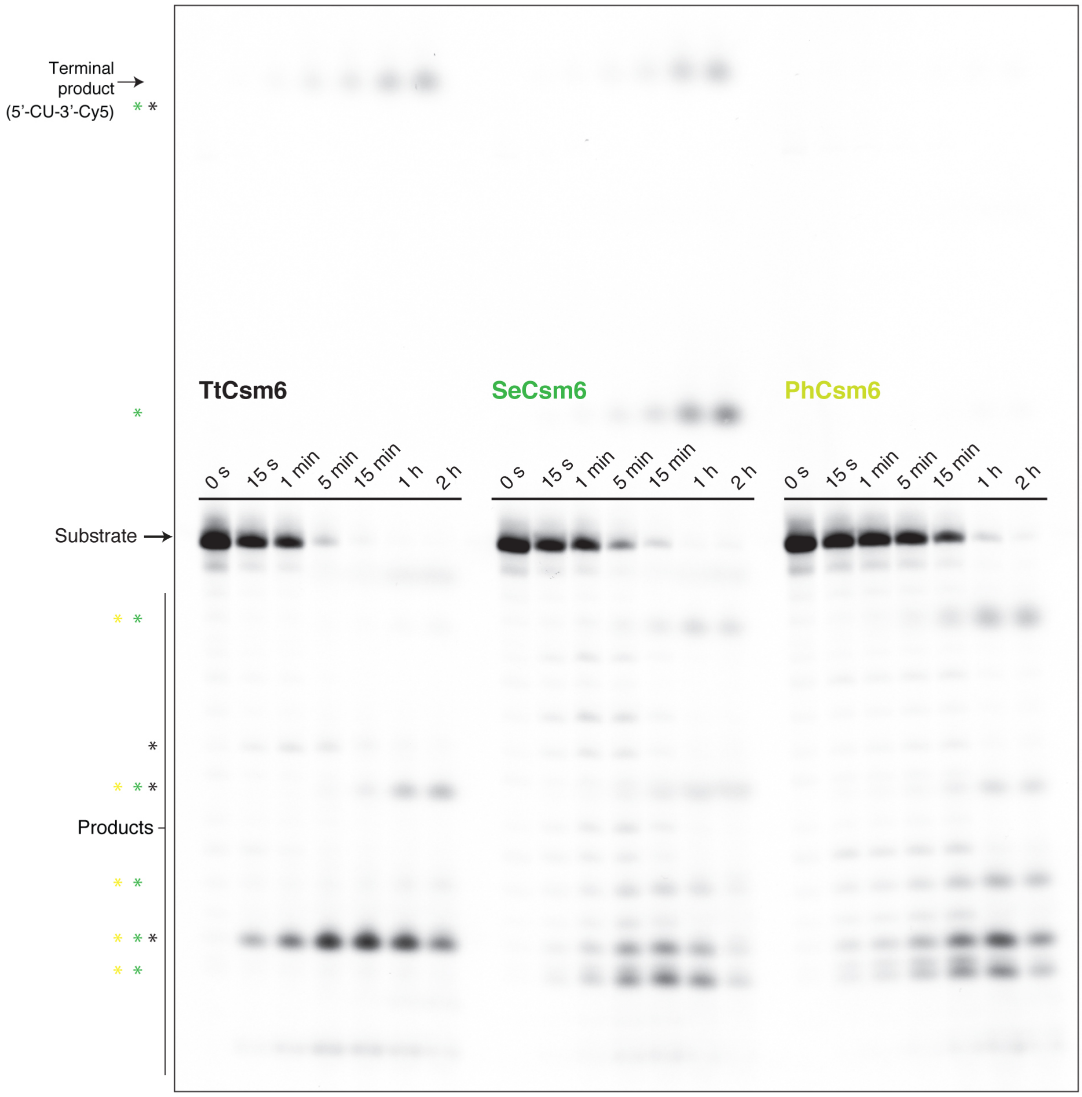

Supplemental Figure 8. The endoribonuclease activity is conserved across Csm6 orthologs.

Nuclease activity assay of TtCsm6 orthologs. The image represents a full-size scan of the image shown in Fig. 3D. The assays were performed using a 24-nt ssRNA substrate labeled with Cy5 at the 3'-end. Reactions were resolved on a 16 % denaturing polyacrylamide gel and visualized using a fluorescence gel scanner. The oligonucleotide fragments produced by each enzyme are indicated by colored asterisks. Note that due to the size and charge of the Cy5 fluorophore, short oligonucleotide products have anomalously low electrophoretic mobility (Killelea et al. 2014).

SUPPLEMENTAL REFERENCES

Killelea T, Saint-Pierre C, Ralec C, Gasparutto D, Henneke G. 2014. Anomalous electrophoretic migration of short oligodeoxynucleotides labelled with 5'-terminal Cy5 dyes. *Electrophoresis* **35**: 1938-1946.

Landau M, Mayrose I, Rosenberg Y, Glaser F, Martz E, Pupko T, Ben-Tal N. 2005. ConSurf 2005: the projection of evolutionary conservation scores of residues on protein structures. *Nucleic acids research* **33**: W299-302.
